# Disentangling the genetic and morphological structure of *Patella candei* complex in Macaronesia (NE Atlantic)

**DOI:** 10.1002/ece3.3121

**Published:** 2017-06-29

**Authors:** Joao Faria, Gustavo M. Martins, Alfonso Pita, Pedro A. Ribeiro, Stephen J. Hawkins, Pablo Presa, Ana I. Neto

**Affiliations:** ^1^ cE3c – Centre for Ecology, Evolution and Environmental Changes/Azorean Biodiversity Group Department of Biology University of Azores Ponta Delgada São Miguel, Azores Portugal; ^2^ Faculty of Marine Sciences – ECIMAT Laboratory of Marine Genetic Resources University of Vigo Vigo Spain; ^3^ Ocean and Earth Science, National Oceanography Centre Southampton Waterfront Campus University of Southampton Southampton UK; ^4^ MARE – Marine and Environmental Sciences Centre and IMAR – Institute of Marine Research Department of Oceanography and Fisheries University of the Azores Horta Portugal; ^5^ The Laboratory Marine Biological Association of UK Plymouth UK

**Keywords:** allopatric speciation, conservation genetics, gene flow barriers, limpets, phenotypic plasticity

## Abstract

The uptake of natural living resources for human consumption has triggered serious changes in the balance of ecosystems. In the archipelagos of Macaronesia (NE Atlantic), limpets have been extensively exploited probably since islands were first colonized. This has led to profound consequences in the dynamics of rocky shore communities. The *Patella candei* complex includes various subspecies of limpets that are ascribed to a particular archipelago and has been the focus of several taxonomic surveys without much agreement. Under a conservational perspective, we apply morphometric and genetic analyses to test subspecies boundaries in *P. candei* and to evaluate its current population connectivity throughout Macaronesia (Azores, Madeira, and Canaries). A highly significant genetic break between archipelagos following isolation by distance was detected (*F*_ST_ = 0.369, *p* < .001). Contrastingly, significant genetic differentiation among islands (i.e., Azores) was absent possibly indicating ongoing gene flow via larval exchange between populations. Significant shell‐shape differences among archipelagos were also detected using both distance‐based and geometric morphometric analyses. Adaptive processes associated with niche differentiation and strong barriers to gene flow among archipelagos may be the mechanisms underlying *P. candei* diversification in Macaronesia. Under the very probable assumption that populations of *P. candei* from each archipelago are geographically and/or ecologically isolated populations, the various subspecies within the *P. candei* complex may be best thought of as true species using the denomination: *P. candei* in Selvagens, *Patella gomesii* in Azores, *Patella ordinaria* in Madeira, and *Patella crenata* for Canaries. This would be in agreement with stock delimitation and units of conservation of *P. candei sensu latu* along Macaronesia.

## 1 INTRODUCTION

Conservation efforts applied to human‐exploited and threatened species require a comprehensive knowledge about population structure and factors that shape differentiation within a species (Lande, 1988). For instance, natural and/or human‐induced barriers across a species distributional range can hasten isolation between populations by restricting the amount of individuals that can freely migrate (Barber, Palumbi, Erdmann, & Moosa, 2000; Kelly & Palumbi, 2010). Such constraints to connectivity can lead to genetically deprived populations that, in the face of intense human exploitation, are likely to succumb and disappear. To overcome isolation, many species have evolved life‐history traits that are able to maximize dispersion and connectivity across large geographical areas (e.g., White, Fotherby, Stephens, & Hoelzel, 2011). For instance, many widely distributed marine organisms with limited adult movement avoid population differentiation by exhibiting large population sizes with high levels of fecundity and by releasing larvae that can potentially disperse in the water column for a considerable amount of time until they reach their final destination (e.g., Faria, Froufe, Tuya, Alexandrino, & Pérez‐Losada, 2013). However, the extent of successful dispersion, which is a major determinant of population dynamics and structure, is a function of multiple and often interacting factors. For instance, an extensively and well‐mixed larval pool does not necessarily lead to widespread connectivity and lack of population genetic structure over large spatial scales (e.g., Keever et al., 2009). The complex interplay between physical processes (e.g., coastal topography, stratified water columns, tidal forces, wind, buoyancy, surface waves, and turbulence) and life‐history traits (e.g., time of spawning, larval behavior, growth and survival rates, pelagic larval duration), often interacting at fine to mesoscales, can result in a broad range of dispersal and metapopulation connectivity patterns (see review in Cowen & Sponaugle, 2009). Similarly, historical events such as past glaciations and changes in sea level can determine the contemporary distribution of populations (e.g., Portnoy et al., 2014).

In the last few decades, genetic methods have become a tool of excellence in investigating population differentiation estimates of a given species across its distributional range. Particularly, high‐resolution nuclear markers such as microsatellites have been widely applied to conservation genetics (Guichoux et al., 2011) and to the identification of populations requiring prioritizing protective measures (e.g., Sandoval‐Castillo & Beheregaray, 2015).

The Macaronesia comprises five NE Atlantic archipelagos: Azores, Madeira, Selvagens, Canaries, and Cape Verde. The region is defined as a biogeographical entity based on the existence of many shared elements in the flora and fauna among archipelagos. All are of volcanic origin and have distinct but fairly recent geological times of origin (Ávila et al., 2016). Patellid limpets inhabiting these archipelagos are considered a valuable resource and have been intensively exploited presumably since islands were first colonized (Côrte‐Real, Hawkins, & Thorpe, 1996; Hawkins, Côrte‐Real, Pannacciulli, Weber, & Bishop, 2000; Santos, Hawkins, Monteiro, Alves, & Isidro, 1995). In most islands, heavy exploitation has led to dramatic decreases in limpet abundances with current populations showing clear signs of over‐exploitation (Martins, Jenkins, Hawkins, Neto, & Thompson, 2008; Martins et al. 2017). Because limpet's grazing activity acts as a key process in shaping the structure and functioning of rocky shore communities (Hawkins & Hartnoll, 1983), the chronic removal of limpets has led to an upward extension of turf‐forming algae (see Boaventura et al., 2002; Martins, Thompson, Neto, Hawkins, & Jenkins, 2010). In fact, under reduced numbers of limpets, algal spores can opportunistically grow to a size that allows them to escape grazing and thus form mature algal patches that are able to persist through time altering the community dynamics and energy flow (Coleman et al., 2006; Martins et al., 2010).

The intertidal limpet *P. candei*, which is exclusive to Macaronesia, occurs on rocky shores from the mid intertidal down to 5 m depth across all archipelagos except in Cape Verde where it is absent (Christiaens, 1973). They are broadcast spawners with fertilization occurring in the water column. According to Martins, Santos, and Hawkins (1987), *P. candei* is a gonochoric species that spawns throughout the year, without synchronized resting periods. While very few studies have sentenced the pelagic larvae duration (PLD) in species of the genus *Patella* (Dodd, 1957; Ribeiro, 2008) with gross estimates ranging from 2 to 32 days depending on the species, temperature, and settlement cues, there is still no available information about the PLD of *P. candei*. Individuals of *P. candei* have a suboval to stellate shell shape and an orange to grayish foot with a thin darker border. Morphological plasticity associated with specific micro‐habitat conditions (i.e., substrate complexity) and environmental variation (i.e., wave exposure) is known to occur in this species (Hawkins, Côrte‐Real, Martins, Santos, & Martins, 1990). For instance, two distinct habitat‐related morphs of *P. candei* are referred for the Azores: the “fly limpet” and the “smooth limpet” (Hawkins et al., 1990). Moreover, the morphological variation associated to each archipelago has led Christiaens (1973) to describe four distinct subspecies within *P. candei* complex: *P. candei gomesii* in Azores, *P. candei ordinaria* in Madeira, *P. candei candei* in Selvagens, and *P. candei crenata* in the Canaries. This classification is not entirely supported by subsequent studies. For instance, Côrte‐Real et al. (1996) found no differences in radular morphology and soft‐body parts among archipelagos but showed that shell shape and allozyme characters from *P. candei* in Azores were clearly distinguished from *P. candei* in the Madeira and the Canaries. Weber and Hawkins (2002) also showed that *P. candei* shell shape could be distinguished among archipelagos and that allozyme retrieved two well‐differentiated groups: *P. candei* from Azores and Selvagens, and *P. candei* from Madeira and Canaries. More recent research, using mtDNA, showed that samples of *P. candei* from the Azores, Madeira, and Desertas (located about 25 km southeast of Madeira Island) form a well‐supported group, while individuals sampled in Selvagens and Canaries always grouped together in a different clade (Sá‐Pinto, Branco, Harris, & Alexandrino, 2005; Sá‐Pinto, Branco, Sayanda, & Alexandrino, 2008). The taxonomic status of Macaronesian limpets thus remains unclear with much controversy as to whether *P. candei* from the different archipelagos should be given a specific status.

In this study, we use morphometric and molecular genetic methods to assess the existence of distinct groups of *P. candei* across Macaronesia, testing the subspecies boundaries within the species. At multiple spatial scales, we evaluate the degree of contemporary connectivity among populations and hypothesize that populations geographically closer to each other are likely more related and connected via larval dispersion. Besides the assessment of genetic diversity and structure of *P. candei* populations across archipelagos, and given the importance of defining conservation units in fisheries planning (Hawkins et al., 2016), we provide discussion and guidance about protective measures of such threatened marine resource, highlighting the importance of considering levels of genetic diversity in populations as well as their uniqueness.

## 2 METHODS

### 2.1 Morphometric analyses

#### 2.1.1 Distance‐based analyses

**Figure 1:**
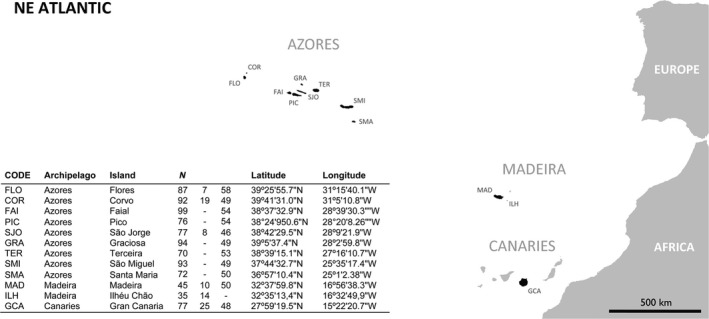
Map of sampling locations for *Patella candei* collected from the Macaronesian archipelagos of Azores, Madeira, and Canaries (NE Atlantic). *N* columns indicate the number of individuals used for distance‐based morphometrics, geometric morphometrics, and genetic analyses, respectively

Individuals of *P. candei* were collected across the Macaronesia archipelagos of Azores, Madeira, and Canarias, in 12 different islands (*n* = 917) during the summer of 2011 (Figure 1). In all islands, individuals were collected on intertidal platforms. The soft tissue of all individuals was carefully removed for genetic analyses, and the shells were marked individually. Shell morphometry was examined using the procedure described in detail by Cabral (2007). In summary, five distances were measured on each shell: shell length (SL), shell maximum width (SW), shell width at apex (SWA), distance from apex to anterior tip (SAA), and shell height (SH). These distances were then used to calculate base ellipticity (BE), base eccentricity (BEC), conicity (CO), and cone eccentricity (CE) for each individual shell (see Cabral, 2007 for further details). Shells were measured using a Vernier caliper with a precision of 0.1 mm. Individuals with signs of shell damage or with clear home fitting deformation were discarded from the analyses. The four variables were inspected for collinearity and removed from the analyses when collinearity was high (*r* > .3).

Examination of the spatial variation in shell morphometry among archipelagos was analyzed using a two‐way fully hierarchical analysis of variance (ANOVA) with the following factors: archipelago (fixed) and island (random and nested within archipelago). Analyses were performed using PERMANOVA+ (Anderson, Gorley, & Clarke, 2008) based on Euclidean distances and using 999 permutations. PERMANOVA is a permutational‐based test and produces results analogous to ANOVA when based on Euclidean distances (Anderson, 2001). This test was preferred over classical ANOVA because it allows the use on unbalanced designs (e.g., different number of islands per archipelago). When needed, SL was used as a covariate in order to adjust for differences in morphometry with animal size. Prior to analysis, PERMDISP was used to test for homogeneity of multivariate dispersions. All analyses were performed on untransformed data. Principal component analysis (PCA) was performed to determine the linear combination of morphometric descriptors that account for the variation in the data. Shell‐shape variation between *P. candei* morphotypes (“fly” and “smooth”) from Azores was also accessed by means of ANOVA. Given the difficulties in sorting all individuals into the “fly” and “smooth” categories, only distinctive shells of each morphotype were used in the analysis (*n* = 346). Note, however, that this criteria may upward bias the degree of differentiation between morphotypes.

#### 2.1.2 Geometric morphometric analyses

A total of 83 *P. candei* collected across archipelagos were used for landmark‐based geometric morphometric analysis (Bookstein, 1991; Figure 1). For imaging, shells were positioned in a light background and digital high‐resolution images of the dorsal surface were captured using a CANON EOS 600D camera mounted on a tripod to maintain the distance for all samples and to ensure that the lens was parallel to the surface examined. The anterior–posterior axis of each specimen was identified using the scars from each individual body left on the ventral surface of the shell. A fan was used to position each specimen along such axis and ensuring that the apex of each shell coincided with the vertical line of the fan (Fig. S1). A *tps* file of each archipelago specimens was created using *tpsUtil,* and *tpsDig2* (Rohlf, 2015) was used to place a total of 37 landmarks on the shell apex and on the intersection of the fan and shell of each sample specimen image. Except for the shell apex landmark, all other points (at the shell border) do not necessarily represent homologous landmarks from a development point of view, but can be used to decompose objectively the shell shape of limpets. These points are referred to as semi‐landmarks and can be used to capture information about curvature (Gunz & Mitteroecker, 2013). Specimens were then aligned using a Generalized Procrustes Analysis (GPA) (Rohlf & Slice, 1990) to remove all the differences due to translation, rotation, and scale (Bookstein, 1991). In this process, semi‐landmarks are allowed to slide along their tangent directions so as to minimize the bending energy between each specimen and the reference form. The resulting aligned Procrustes coordinates represent the shape of each specimen. For more details on geometric morphometric methodologies using landmarks, see Bookstein, 1991; Zelditch, Swiderski, & Sheets, 2012; Adams, Rohlf, & Slice, 2013;. Centroid size (CS), given by the square root of the sum of squared distances of a set of landmarks from their centroid, was also calculated (Rohlf & Slice, 1990). A Procrustes permutation analysis of variance (Procrustes ANOVA) performed with a residual randomization permutation procedure (Adams, Collyer, Kaliontzopoulou, & Sherratt, 2016; Collyer, Sekora, & Adams, 2015) was used to determine patterns of shell shape variation between archipelagos. The aim is to test groups (archipelagos) considering the influence of phenotypic change associated with body size (i.e., body size was calculated from landmark configurations as centroid size). Actually, the Procrustes ANOVA between shape and size, using CS is a way to assess interspecific allometry (Villegas, Feliciangeli, & Dujardin, 2002). Because allometry was detected among archipelagos (see section 2.1), the full dataset was divided in two: one with small limpets (SMALL) and one with big limpets (BIG). Principal components analysis (PCA) was used to provide a graphical depiction of patterns of shape variation across the two datasets. Thin plate splines were used to provide a visual representation of the shape changes between each group mean and overall consensus configuration. All analyses and graphical representations were performed in R (R Core Team 2014) using the packages GEOMORPH (Adams & Otarola‐Castillo, 2013). Geometric morphometric analysis was also used to determine shell shape variation between the “fly” and “smooth” *P. candei* morphotypes from Azores (*n* = 23 and *n* = 34, respectively).

### 2.2 Genetic analysis

#### 2.2.1 Sampling and laboratory protocols

A total of 560 individuals of *Patella candei* from the three archipelagos were used for genetic analysis (Figure 1). Upon collection, limpets were preserved in 96% ethanol and frozen for later processing. At the laboratory, samples were subject to DNA extraction from the foot muscle tissue using the E.Z.N.A. Mollusc DNA extraction kit and following the manufacturer’ instructions. The quality and quantity of DNA extractions were assessed using a Nanodrop spectrophotometer (Thermo Scientific). All individuals were genotyped at 12 microsatellite loci using the primer pairs and following the amplification protocol described in Faria et al. (2016). Briefly, microsatellites were amplified in three distinct multiplex PCRs (PcaMix1: loci *CAN18*,* CAN25*,* CAN27*,* CAN53*; PcaMix2: loci *CAN9*,* CAN26*,* CAN32*,* CAN40*; PcaMix3: loci *CAN23*,* CAN33*,* CAN56*,* CAN60*) on 10 μl reactions containing ~30 ng DNA template, 1 × Qiagen^TM^ Multiplex PCR Kit, 0.5–1.2 μmol/L of each primer and ddH_2_O. Genotyping was performed on an ABI 3730 (Applied Biosystems) automated DNA sequencer using an internal size standard (GeneScan^TM^ 500Liz^®^, Applied Biosystems) for accurate sizing and GENEMAPPER^TM^ v.4.2 (Applied Biosystems) was used for allele calling.

#### 2.2.2 Genetic variation

Genetic diversity estimates such as allele frequencies and observed and expected heterozygosities (*H*_O_ and *H*_E_) were estimated in GenAlEx v.6.4.1 (Peakall & Smouse, 2006). The fixation index (*F*_IS_), linkage disequilibrium, and deviations from the Hardy–Weinberg equilibrium (HWE) were tested in GENEPOP v.4.2 (Raymond & Rousset, 1995). Allelic richness [Ar(g)] and private allele richness [Ap(g)] were estimated using the rarefaction method implemented in ADZE v.1.0 (Szpiech, Jakobsson, & Rosenberg, 2008). Whenever needed, the false discovery rate (FDR) control was employed to account for multiple testing (Verhoeven, Simonsen, & Mcintyre, 2005). The presence and frequency of null alleles was tested for each locus using MICROCHECKER v.2.2.3 (Van Oosterhout, Hutchinson, Wills, & Shipley, 2004) and FREENA (Chapuis & Estoup, 2007), respectively.

#### 2.2.3 Genetic differentiation and population structure

Pairwise *F*_ST_ estimates among populations were calculated using FSTAT v.2.9.3 (Goudet, 1995), and departures of *F*_ST_ from the null hypothesis of panmixia were evaluated via a permutation test (1,000 iterations). The effect of null alleles in *F*_ST_ estimates was assessed by comparing *F*_ST_ before and after correction for null alleles using the excluding null alleles (ENA) method implemented in FREENA. Genetic differentiation between populations was also determined using the *D*_est_ estimator (Jost, 2008) implemented in the R package DEMETICS v.0.8.4 (Gerlach, Jueterbock, Kraemer, Deppermann, & Harmand, 2010), and *p*‐values were estimated by bootstrap analysis (1,000 replicates). For all analyses involving multiple tests, significance levels were adjusted by the FDR method.

The model‐based approach implemented in STRUCTURE v.2.3.3 (Pritchard, Stephens, & Donnelly, 2000) was used to identify the most likely number of populations (*K*) and assign individuals to genetic clusters. Assignment is conducted in ways that minimize deviations from Hardy–Weinberg and linkage equilibrium within each cluster. No particular population structure was assumed a priori (LOCPRIOR = 0), and ten independent runs were carried out for each value of *K* (1–11). Length of the burn‐in period was set to 1 × 10^5^ followed by 5 × 10^5^ Markov chain Monte Carlo (MCMC) iterations. Correlated allele frequencies and admixed populations were assumed. Modifications in such parameters produced consistency and did not change the final results. Selection of the most likely number of genetic clusters (*K*) was based on checking the posterior probability of the data for a given *K* (Pritchard et al., 2000) and also by looking at the second‐order rate of change in probability between successive *K* values as described in Evanno, Regnaut, and Goudet (2005) and implemented in STRUCTURE HARVESTER (Earl & vonHoldt, 2012). In systems with hierarchical population structure, STRUCTURE typically best resolves the highest level of population subdivision (Evanno et al., 2005). Thus, in order to resolve lower levels of subdivision, structure analyses were also conducted separately for each cluster identified. Therefore, two additional STRUCTURE analyses using the same settings were used to identify potential within‐cluster structure. The best *K* was determined as previously described.

A discriminant analysis of principal components (DAPC) was also performed to identify and describe clusters of genetically related individuals (Jombart, Devillard, & Balloux, 2010). DAPC has been shown to perform generally better than STRUCTURE at characterizing population subdivision (Jombart et al., 2010). DAPC is a multivariate analysis that integrates principal component analysis (PCA) together with discriminant analysis to summarize genetic differentiation between groups. DAPC is free of assumptions about Hardy–Weinberg equilibrium or linkage disequilibrium and provides graphical representation of divergence among populations. DAPC was performed with and without using prior group information using the R package ADEGENET (Jombart, 2008). All STRUCTURE and DAPC analyses were conducted upon removal of nonamplifying loci.

Tests for genetic differentiation among archipelagos were also conducted using analysis of molecular variation (AMOVA) in ARLEQUIN v.3.5.1.3 (Excoffier & Lischer, 2010). Genetic variation among archipelagos (F_CT_), among populations within archipelagos (*F*_SC_) and within populations (*F*_ST_) was assessed, and significance of *F*‐statistics was tested using 10,000 permutations. Estimates of genetic differentiation were also determined among the “fly” and “smooth” morphotypes of *P. candei* from Azores.

#### 2.2.4 Isolation by distance and gene flow

To test for isolation by distance (Wright, 1943), linearized *F*_ST_ transformation (*F*_ST_/[1 − *F*_ST_]) was regressed onto the natural log of geographic distances (GD; Rousset, 1997). Regression with GD was also performed with the differentiation estimator *D*_est_ matrix. Regression analyses were performed in R and tested for significance with a Mantel permutation procedure. Moreover, given the heterogeneous nature of samples, the Monmonier's maximum difference algorithm implemented in BARRIER v.2.2 was used to highlight geographical features associated with genetic discontinuities among populations (see Manni et al. 2004 for method details). Analyses were conducted using pairwise *F*_ST_ values, and statistical confidence for each identified barrier was evaluated using 100 bootstrap replicates that were simulated using the package diveRsity in R. Analyses were also conducted separately for each amplifying microsatellite locus.

Recent migration rates (*m*) among populations/clusters identified in STRUCTURE were estimated using the Bayesian multilocus genotyping procedure implemented in BAYESASS v.3.0. (Wilson & Rannala, 2003). Analyses were only conducted among archipelagos due to the lower accuracy of BAYESASS when migration rates are high and genetic differentiation is low (see section 2.1) (Faubet, Waples, & Gaggiotti, 2007; Meirmans, 2014). The program was run for 3 × 10^6^ MCMC iterations with sampling at every 1,000 iterations, of which 10^6^ iterations were discarded as burn‐in. Delta values for allele frequency, migration rate, and inbreeding were adjusted so that the accepted numbers of changes were 40%–60% of the total number of iterations. Ten MCMC runs with different initial seeds were carried out in order to maximize convergence and mixing. The Bayesian deviance was used as an optimality criterion to find the run with the best fit (Faubet et al., 2007). Deviance was calculated from the trace file using the R‐script provided by Meirmans (2014).

Contemporary gene flow was also estimated using the F‐model on BIMr program (Faubet & Gaggiotti, 2008). This software can estimate migration rates and detect migrants (within the last generation) at a lower level of population differentiation compared to BAYESASS (Faubet & Gaggiotti, 2008). In addition, BIMr can identify the environmental factors that are more likely to explain the observed patterns using a generalized linear model. The method employs a Bayesian approach and Markov chain Monte Carlo (MCMC) techniques to make inferences of recent gene flows in subdivided populations (Faubet & Gaggiotti, 2008). Preliminary trials included all populations but because population‐specific *F*_ST_ values below 0.01 can be problematic for parameter estimation, analyses were performed on samples grouped according to the Bayesian clustering analyses results. Also, analyses were conducted with and without removing loci that failed to amplify in some populations and/or exhibited null alleles. Often considered one of the main factors in determining gene flow in many species, the geographic distance between samples was included as the environmental variable. A total of 20 independent replicate runs were performed. Each MCMC was run for a total of 3.53 × 10^6^ iterations, which included 30 short pilot runs of 1,000 iterations each in an effort to obtain acceptance rates between 25% and 45%. The next 15 × 10^5^ iterations were discarded as burn‐in, and a total of 20,000 samples were collected from each of the 20 replicates using a thinning interval of 100 iterations, using default settings. The posterior probabilities were evaluated for the run with the lowest Bayesian deviance (given by the assignment component of the total deviance: *D*_assign_) (Faubet & Gaggiotti, 2008; Faubet et al., 2007). The mean, mode (point estimate), and 95% highest posterior density intervals (HPDI) for migration rates were recorded.

## 3 RESULTS

### 3.1 Distance‐based morphometrics

**Figure 2:**
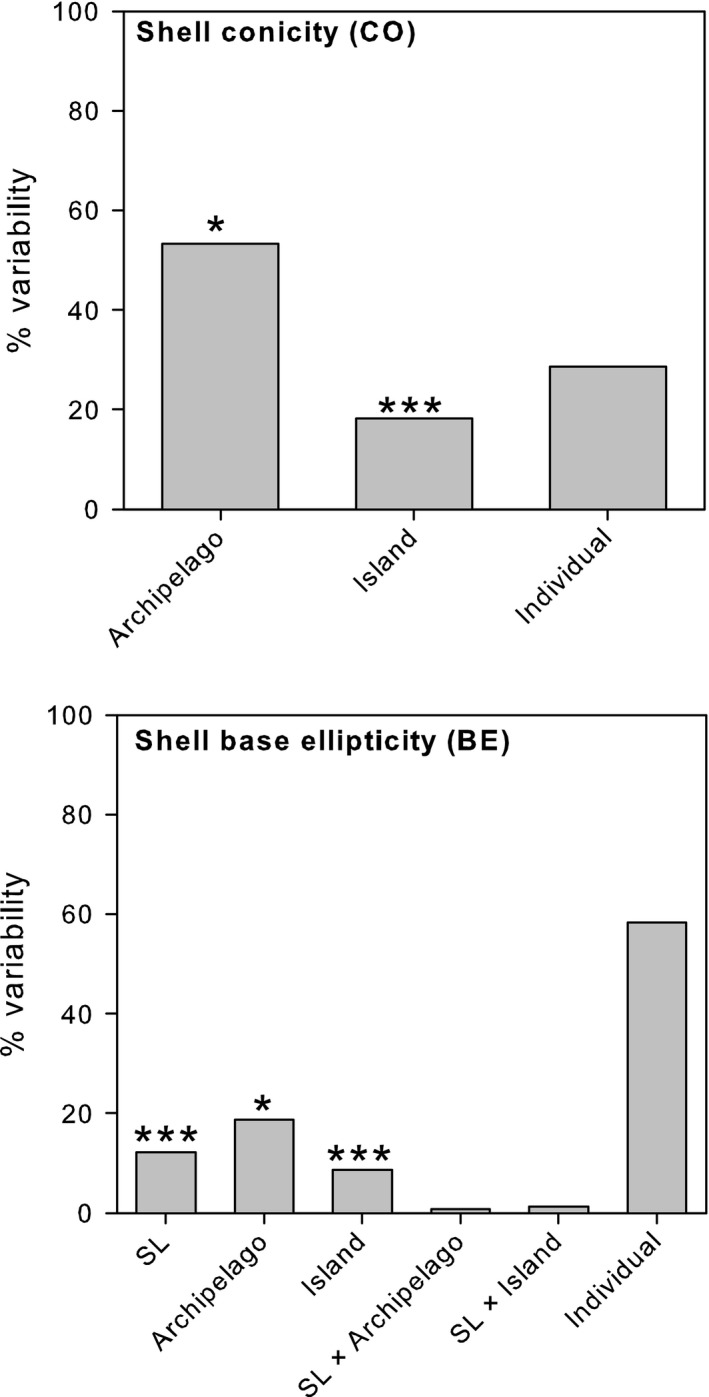
Components of variability for shell conicity (CO) and shell base ellipticity (BE) in *Patella candei* across archipelagos; SL stands for shell length. Significance: *p < .05, ***p < .001 (see Tables S3 and S4 for ANOVA terms)

**Figure 3:**
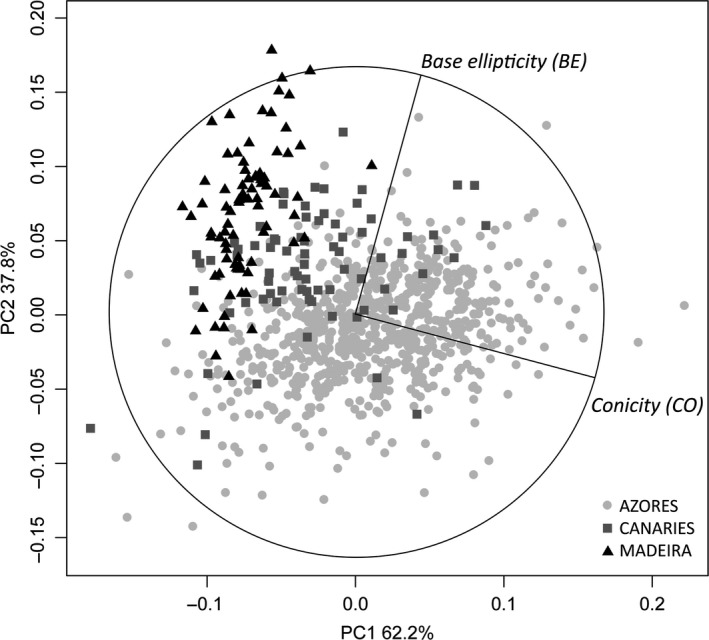
Principal component plot for shell morphometric descriptors, conicity (CO) and base ellipticity (BE), in *Patella candei* from the Macaronesian archipelagos of Azores (*N* = 760), Madeira (*N* = 80), and Canaries (*N* = 77); eigenvector PC1: BE (0.258), CO (0.966); PC2: BE (0.966), CO (−0.258)

Shell samples of *P. candei* ranged in size (SL) between 1.35 and 6.35 cm, with a mean size of 3.25 ± 0.03 cm (mean ± *SE*). Although variable across islands (ANOVA *p* < .001), mean SL did not differ among archipelagos (ANOVA *p* > .05, Fig. S2, Table S1) but was significantly correlated with the remaining distance measures (SW, SWA, SAA, and SH) and also with BE (Table S2). Hence, only SL was used for analyses and considered as a covariate in the analysis of spatial variation of BE. Among the morphometric descriptors, conicity (CO) was positively correlated with BEC and CE and was therefore selected for analysis together with BE. Significant variation in the shape, as given by analyses of the descriptors CO and BE, was found at the scale of islands and among archipelagos (ANOVA *p* < .05, Tables S3 and S4). Variation in shell BE among archipelagos, although affected by size (SL), was relatively higher than variation among islands (Figure 2). Similarly, for conicity, the largest proportion of the variability was found at the scale of archipelagos (Figure 2). The first principal component (PC1) described 62.2% of the total variation, with the remaining variation (%) being accounted by the second principal component (PC2). The most important variable integrated by the first and second components was conicity and BE, respectively. The PCA showed that shell conicity can better distinguished shells from different archipelagos, whereas shell BE was mostly associated with differences within archipelagos (Figure 3). Significant shell shape variation was also detected between the two *P. candei* morphotypes in Azores (ANOVA, *p* < .01). Differences were only detected for shell conicity, with “fly limpets” being more conical then “smooth limpets.”

### 3.2 Geometric morphometrics

**Figure 4:**
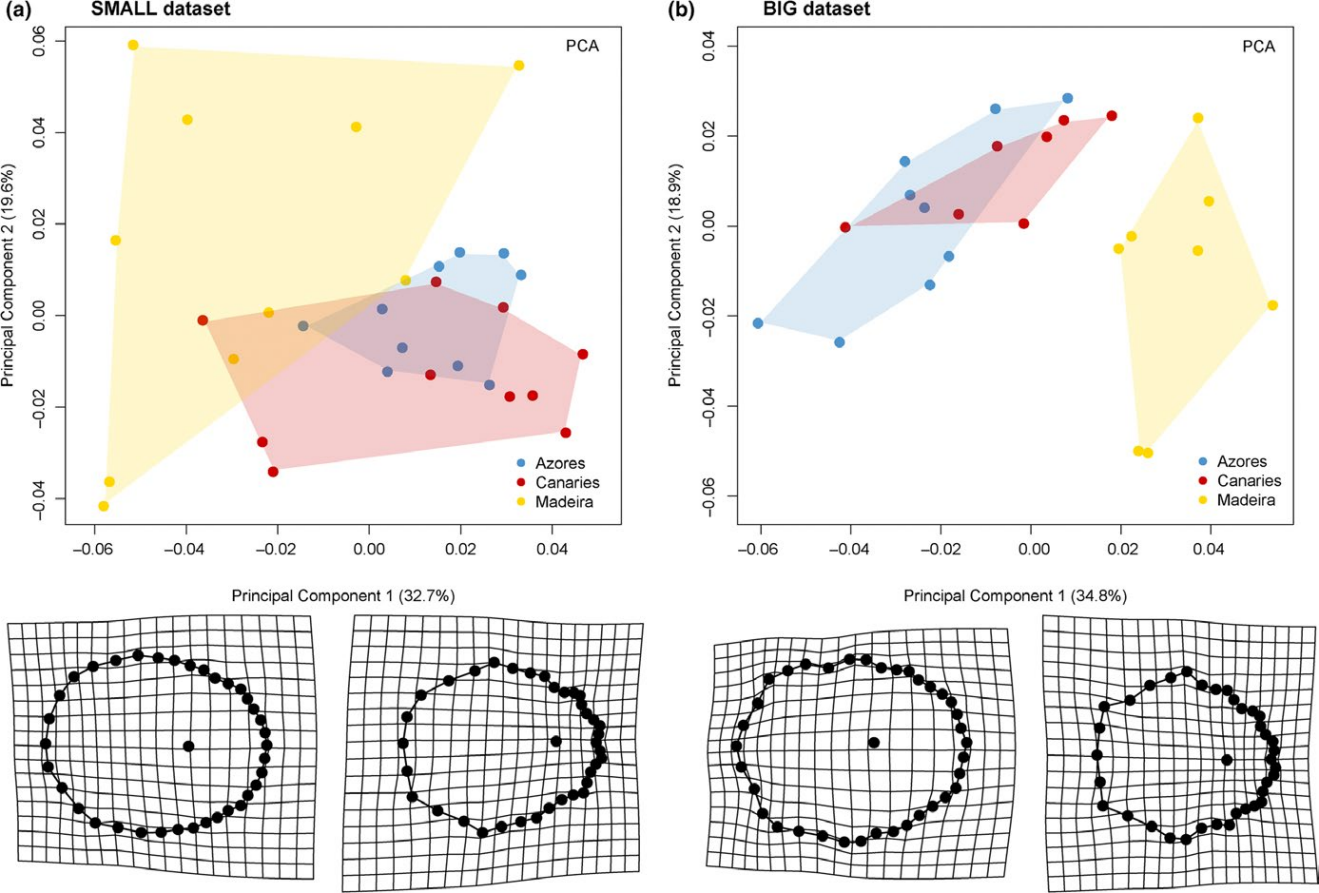
Principal component analyses of Procrustes coordinates derived from the first two principal components (PC1 and PC2) for the (a) SMALL dataset and (b) BIG dataset. Convex hulls are drawn to show the area of the morphospace occupied by each archipelago; the thin‐plate spline deformation grids display the shape of specimens at the ends of the range of variability along each PC1

The Procrustes ANOVA analysis on the full shell shape dataset revealed a significant interaction between archipelago and size, indicating an allometric growth in *P. candei* (Table 1). Moreover, the null hypothesis for common allometries (parallel slopes) among archipelagos was rejected (*F* = 5.364, *p* < .001). The amount of shape change per unit of size change differed among archipelagos and was greater in Azores (indicated by the lengths of slope vectors) (Table 1; Fig. S3). Yet, shape trajectories and the way shapes change were only significantly different between Canaries and Madeira (indicated by the angles between slope vectors) (Table 1; Fig. S3). This corresponds to contrasting local deformations in particular parts of the landmark configuration associated with size change in these two archipelagos (e.g., note the changes in the shell apex landmark in Madeira and Canaries; Fig. S4). Significant differences in shell shape unrelated to size were detected among archipelagos for the two subsets (SMALL and BIG; Table S5). For both datasets, pairwise comparisons showed that Azorean and Canaries shells could not be distinguished (Table S5). The first two principal components of the Procrustes shape variables for each dataset accounted for 52 and 53% of the total sample variation, respectively (Figure 4). A generalized overlapping in the scatter of data was found, mostly between Azores and Canaries samples. Intraspecific variance was greatest in smaller shells from Madeira and Canaries, with individuals from these archipelagos occupying a much wider range of shape space than samples from Azores. Deformation grids for both SMALL and BIG datasets indicate that shell goes from a clear round shape to a more ridged and pointy look‐alike shape along CV1 (left to right) (Figure 4). On the same direction, the shell apex gets closer to the anterior margin of the shell. Similarly, the anterior end of the shell gets narrower along such axis. These shape changes are mostly associated and illustrate shell shape differences between Azores/Canaries and the Madeira samples. Whereas Azorean shells are oval with a smoother margin, the Canaries samples exhibit some ridges along their shell border. The pentagon look‐alike shape of *P. candei* in Madeira stands out from the remaining archipelagos (Fig. S5). As for shell shape variation between *P. candei* morphotypes (“fly” and “smooth”) from Azores, the Procrustes ANOVA analysis revealed a significant interaction between morphotype and size, which is indicative of allometric growth. The null hypothesis for common allometries (parallel slopes) among morphotypes was rejected (*F* = 7.459, *p* < .01), and differences were detected in the amount of shape change per unit of covariate change (size) between morphotypes; shape change per size unit is higher in “fly” limpets (Table S6). Overall shape of *P. candei* in Azores is oval for both morphotypes but the shell apex in “fly” limpets tends to get closer to the anterior margin of the shell (Fig. S6).

**Table 1:** Procrustes ANOVA examining differences in patterns of shell shape variation among archipelagos (10,000 random permutations)

	*df*	*R* ^2^	*Z*	*F*
SIZE log(CS)	1	.136	9.814	15.480***
ARCHIPELAGO	2	.149	6.695	8.502***
SIZE log (CS) × ARCHIPELAGO	2	.039	2.146	2.226***
Total	82			
Slope pairwise comparison	AZORES	CANARIES	MADEIRA	
AZORES	–	0.121*	0.118*	
CANARIES	47.4	–	0.097*	
MADEIRA	45.8	50.3**	–	

Centroid size (CS) was used as a covariate. Slope pairwise comparisons among archipelagos are shown; contrasts in slope vector length and angles between slope vectors are shown in upper and lower diagonal, respectively.

Effect sizes (Z) are standard deviations of the observed size.

*p < .05, **p < .01, ***p < .001.

### 3.3 Genetic analysis

A total of 138 alleles were observed across the 12 loci examined, ranging from six in CAN26 and CAN56 to 22 alleles in CAN18 (Table S7). Five loci failed to amplify in individuals from Madeira and Canaries: Loci CAN9, CAN26, and CAN33 did not amplify at all in these two populations and two additional loci failed to amplify in more than 30% of both samples (Table S8). Since microsatellite markers were developed using *P. candei* from the Azores (see Faria et al., 2016), such amplification failure suggests a high genetic differentiation between Azores and the remaining archipelagos. Multilocus mean allelic richness with rarefaction was similar across populations and ranged from 3.6 (GCA) and 4.7 (FAI). Mean number of private alleles was greater in the MAD population (Ap[30] = 1.14). Observed heterozygosity frequencies (H_O_) were relatively low and ranged from 0.157 to 0.364, while expected (*H*_E_) heterozygosity frequencies ranged from 0.304 to 0.484 (Table S7). No significant linkage disequilibrium was detected for any pairs of loci. Except for loci CAN23 and CAN53, all other loci deviated from Hardy–Weinberg equilibrium (HWE). Significant locus‐specific inbreeding coefficients (*F*_IS_) ranged from 0.073 to 0.746 denoting a heterozygous deficit in such loci. Overall, significant *F*_IS_ values were often ascribed with the presence of null alleles. To check for any bias in the results, loci with a presence of null alleles >10% were removed from the analysis. Such removal did not affect genetic differentiation results (data not shown) and unless stated otherwise, all loci were included in subsequent genetic analyses. In fact, the influence of null alleles has been shown to be marginal when compared to other factors such as number of loci and strength of population differentiation (Carlsson, 2008). Pairwise comparisons of *F*_ST_ and *D*_est_ indicated high and significant genetic differences among archipelagos but not within archipelago (i.e., between islands in Azores) (Table 2). Both indices were highly correlated (Pearson's correlation 0.99, *p* < .001) (Fig. S7). Furthermore, *F*_ST_ values before and after correction for null alleles using the ENA method did not differ considerably (Table S9).

**Table 2:** Pairwise estimates of *F*_ST‐FREENA_ (below diagonal) and Jost's *D*_est_ (above diagonal) between all populations sampled for *Patella candei* (see Figure 1 for population codes)

	FLO	COR	FAI	PIC	SJO	GRA	TER	SMI	SMA	MAD	GCA
FLO	–	0.020	0.010	0.000	0.018	0.005	0.002	0.022	0.033	**0.505**	**0.594**
COR	0.008	–	0.006	0.016	0.009	0.008	0.005	0.006	0.010	**0.492**	**0.588**
FAI	0.004	0.002	–	0.007	0.007	0.005	0.014	0.010	0.015	**0.481**	**0.577**
PIC	0.000	0.008	0.000	–	0.004	0.000	0.003	0.004	0.008	**0.504**	**0.596**
SJO	0.004	0.004	0.000	0.001	–	0.000	0.013	0.001	0.006	**0.474**	**0.587**
GRA	0.000	0.004	0.000	0.000	0.000	–	0.008	0.004	0.002	**0.494**	**0.588**
TER	0.005	0.010	0.011	0.009	0.009	0.006	–	0.014	0.020	**0.500**	**0.597**
SMI	0.005	0.007	0.000	0.000	0.000	0.000	0.009	–	0.000	**0.493**	**0.594**
SMA	0.006	0.008	0.006	0.001	0.002	0.000	0.010	0.000	–	**0.499**	**0.597**
MAD	**0.319**	**0.307**	**0.296**	**0.308**	**0.286**	**0.308**	**0.336**	**0.314**	**0.323**	–	**0.203**
GCA	**0.388**	**0.376**	**0.360**	**0.373**	**0.361**	**0.377**	**0.405**	**0.385**	**0.392**	**0.127**	–

Significant values after FDR correction are shown in bold.

**Figure 5:**
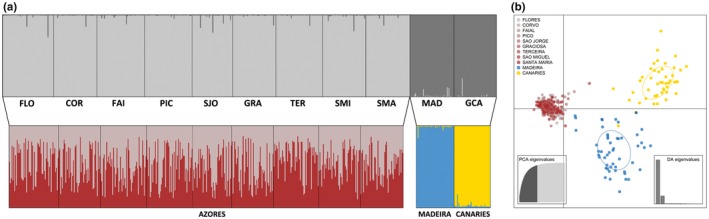
Genetic differentiation in *Patella candei* using (a) STRUCTURE analyses: *K* = 2 using all populations (upper plot); *K* = 2 for populations from Azores and also *K* = 2 between Madeira and Canaries (bottom plot). Each individual is represented by a vertical bar in *K* colored segments with the length of each bar being proportional to the estimated membership coefficient. Black lines separate populations from different geographic regions (see Figure 1 for population codes); and (b) DAPC analysis

**Figure 6:**
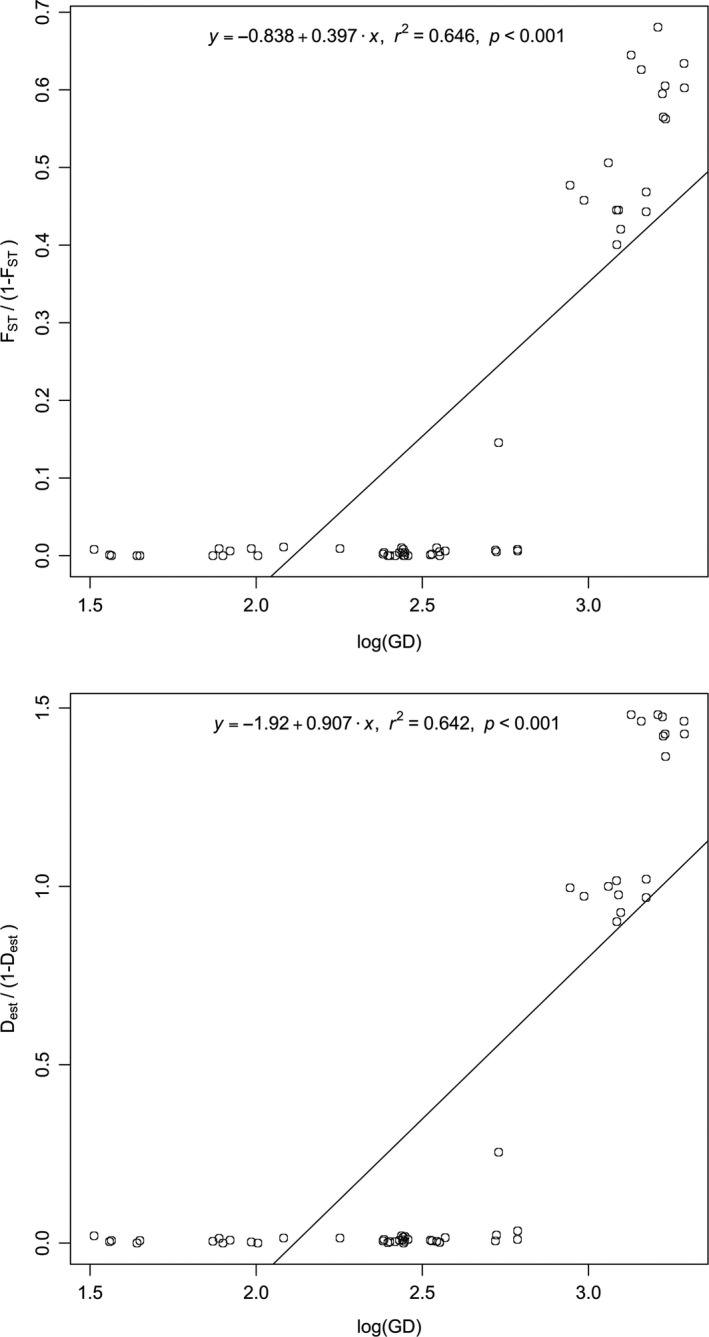
Regression between genetic distances *F*_ST_/(1 − *F*_ST_) (top plot) and *D*_est_ (bottom plot), with natural log geographical distances. Whereas a strong signal of IBD is observed among archipelagos, basal pairwise points in both graphs indicate that genetic differentiation between geographically separated islands within archipelago is absent (i.e., Azores)

**Figure 7:**
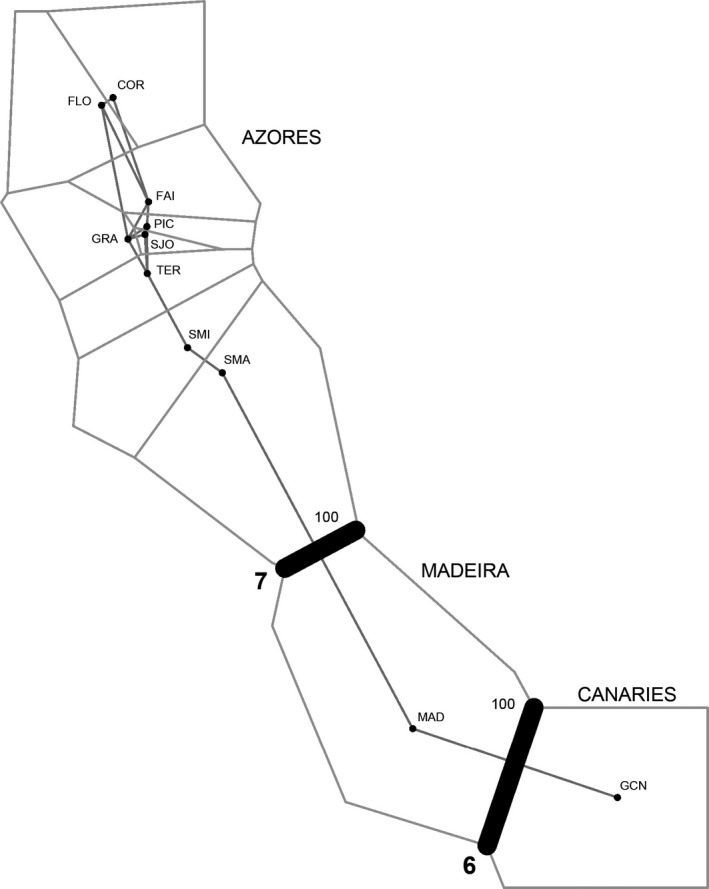
Areas of genetic discontinuity identified with BARRIER using the Monmonier's algorithm. Barriers to gene flow are indicated by thick black lines; the smaller adjacent numbers relate to the proportion of times the barrier was observed across 100 bootstrap replicates; bolded figures indicate the number of loci out of nine supporting the observed barriers. Only those barriers supported by more than half the loci set and high bootstrap values (>50%) are shown

The STRUCTURE and DAPC analyses provided support for the genetic differentiation indicated by *F*_ST_ and *D*_est_. In fact, the genetic structure inferred from the 560 individuals of *P. candei* and the 12 microsatellite loci using the Bayesian model‐based clustering algorithm, and the model‐free DAPC clustering algorithm, provided similar results (Figure 5). Two clusters (*K* = 2) were identified when considering all locations, with Azores samples being separated from the Madeira and Canaries populations. Similarly, two well‐defined clusters were retrieved on the Madeira and Canaries assignment analysis. Both STRUCTURE and DAPC also suggested population homogeneity for *P. candei* in Azores (*K* = 1). In this case, for the STRUCTURE analysis, the estimated membership of individuals to any given cluster was roughly symmetric (~1/*K* in each population), indicating that individuals in Azores are widely admixed and belong to a single panmictic population (Figure 5). Results from the Bayesian clustering analysis and DAPC were consistent with those obtained using AMOVA, which detected significant genetic differences among archipelagos. According to the AMOVA, 36.6% of the total genetic variation was found among archipelagos (*p* < .001), 0.2% among populations of the same archipelago (*p* = .268), and 63.1% within populations (*p* < .001). Furthermore, a significant positive relationship was observed between genetic differentiation estimators (*F*_ST_ and *D*_est_) and geographic distance (Figure 6). However, such relationship (IBD) is only endorsed among archipelagos and not within archipelago (i.e., populations from Azores are genetically undistinguished) (Figure 6). The Monmonier's algorithm in BARRIER revealed the existence of strong spatial barriers to gene flow among archipelagos (Figure 7). No genetic differentiation was found between *P. candei* morphotypes from Azores (*F*_ST‐FREENA_ = 0.001 and *D*_est_ = 0.002, both nonsignificant; STRUCTURE: best *K* = 1).

The results of the migration rates estimated in BAYESASS suggest a consistent restriction in contemporary gene flow between archipelagos (Table 3). Yet, from the individual assignments, two individuals sampled in Madeira had posterior probability estimates (0.13 and 0.44, respectively) as second‐generation migrants from Canaries. One individual sampled in the Canaries had a probability of 0.24 to be a first‐generation migrant from Madeira (Table S10). Estimates of current gene flow rates from BIMr for populations of *P. candei* were consistent across all preliminary trials and runs and showed mean values very close to 0 (Table 3). Such small estimates suggest effectively the absence of contemporary gene exchange among archipelagos. Moreover, the highest posterior probability was assigned to the model excluding distance as a factor (*P*[none] = 57.3; *P*[distance] = 42.7). The observed migration rates were independent of geographic distance as migration between archipelagos was similar and virtually absent.

**Table 3:** Contemporary gene flow in *Patella candei* between archipelagos as depicted with the average posterior distribution of migration rates from (a) BAYESASS (and 95% CI) and (b) BIMr (95% HPDI)

From/into	Azores	Madeira	Canaries
(a) BAYESASS v.3.0			
AZORES	0.999 (0.997–1.000)	0.006 (0.000–0.019)	0.007 (0.000–0.020)
MADEIRA	0.001 (0.000–0.002)	0.979 (0.953–1.000)	0.009 (0.000–0.024)
CANARIES	0.001 (0.000–0.002)	0.015 (0.000–0.037)	0.985 (0.963–1.000)
(b) BIMr			
AZORES	1.000 (0.993–1.000)	0.000 (10^−5^–0.003)	0.000 (10^−17^–0.005)
MADEIRA	0.000 [0.000–0.051)	0.999 (0.910–0.996)	0.000 (0.000–0.082)
CANARIES	0.000 [0.000–0.0298)	0.000 (10^–5^–0.034)	1.000 (0.964–0.998)

## 4 DISCUSSION

Here, we investigate current signs of population differentiation and connectivity in the *P. candei* complex across Macaronesia (NE Atlantic). Our results revealed highly structured populations among archipelagos, which are likely associated with strong barriers to gene flow. Although isolation by distance (IBD) was detected among archipelagos, connectivity within archipelago (i.e., Azores) does not follow IBD, with genetic homogeneity among populations (i.e., islands) being maintained possibly via broad larval exchange. Moreover, shell shape differences among archipelagos were also detected and are likely the consequence of mixed effects of historical vicariance and recolonization events, genetic drift, and local adaptations.

Limitations to this study include the lack of samples from some Macaronesia islands (i.e., no samples from Selvagens and single samples from Canaries and Madeira), so that results among archipelagos cannot be fully explored in comparison with patterns within archipelagos. Despite this shortcome, the inclusion of samples from Madeira and Canaries allowed showing that the *Patella candei* complex across Macaronesia is highly differentiated and that each subspecies (or species) endorsed to a given archipelago should be treated as a single conservation unit.

### 4.1 Shell shape variation between archipelagos

There have been few attempts to describe and distinguish limpet species and/or morphotypes using shell morphometry (e.g., Cabral, 2007; Denny, 2000). The difficulties of such methods rely on the fact that limpet shells have a suboval shape without any clear external homologous landmarks (except for the shell apex) or readily identifiable morphological features. Furthermore, due to the strong influence of certain environmental factors (i.e., wave exposure, substrate complexity, predation) many organisms, including limpets, exhibit high morphological plasticity (Branch & Marsh, 1978; Guerra‐Varela et al., 2009; Harley, Denny, Mach, & Miller, 2009; Lowell, 1986; Sokolova & Berger, 2000). The advent of modern techniques such as geometric morphometrics has furthered the ability of researchers to differentiate species and or specimens based on morphological characters (e.g., Baylac, Villemant, & Simbolotti, 2003; Davis, Douglas, Collyer, & Douglas, 2016; Ruane, 2015). In such sense, whereas distance‐based methods are considered rather more simplistic in detecting shape differences between samples, the geometric analysis allows a more detailed recognition and assessment of how such shape varies with shell growth. Despite intrinsic different, generally, both methods allowed to distinguish *P. candei* morphotypes among and within archipelagos. The differences found between both methods are likely associated to (1) the fact that distance‐based data contain relatively little information about shape because many of the measurements overlap and/or are correlated to each other, and shape can only be derived from ratios among particular measurements and (2) the inclusion of shell height to the distance‐based method provides a third dimension, which is absent in geometric morphometrics that only considers a two‐dimensional reduction of shape in current analyses. Depicting the results, distance‐based methods show that samples from Azores exhibit a more conical and elliptical shell shape compared to samples from the Canaries and Madeira, with the later descriptor being influenced by size. In fact, shell shape allometries derived from geometric morphometrics differ among archipelagos, and globally, differences are mostly found between Madeira samples and the remaining archipelagos; both small and large limpets from Azores and Canaries are more similar between them than with samples from Madeira. Whereas genetic data suggest a closer relationship between *P. candei* from Madeira and Canaries populations (see Sections 3 and 4 below), shell shape differences are more thinned between *P. candei* from Azores and Canaries. This variation in morphology does not need to be necessarily consistent with genetic variation, especially because neutral markers such as microsatellites can be subject to distinct evolutionary forces than selected loci (McKay & Latta, 2002).

Shell shape variation in *P. candei* among archipelagos seems to be associated with mixed effects of ancient vicariance events, genetic drift and particular local adaptations under restrictive gene flow that acted together to produce such dissimilarities. For instance, the genetic stochasticity associated with distinct evolutionary histories such as time of colonization in each archipelago and/or specific retraction and expansion demographic events under limited gene flow may have contributed to the observed morphological variation. These mechanisms are likely responsible for the occurrence of several patellid species with considerable differentiation in shell shape on similar habitats across Macaronesia. For example, in Madeira, *P. candei* and *Patella piperata* have an overlapping intertidal distribution, even though the shell of *P. piperata* is more round shaped and exhibits small black granules along its shell. Historical and/or contemporary diverging local selection pressures, and subtle differences in temperatures, hydrodynamic forces, substrate composition, community assemblages, and available competitors among archipelagos are also likely to have played a key role in determining variation in limpet shell shape on such remote islands. Moreover, the adaptive phenotypic plasticity associated with multiple environmental conditions, which is common in many intertidal molluscs (e.g., De Wolf, Backeljau, Medeiros, & Verhagen, 1997; Trussell, 2000), can also determine geographic variation. Within the Azorean archipelago, such phenotypic plasticity is evident in the two well‐recognized habitat morphs: The “fly limpet” which is highly conical and commonly found upper on the shore, mostly on more rugose surfaces; and the “smooth limpet,” which is more flattened and associated to surfaces highly exposed to hydrodynamic forces (Hawkins et al., 1990). Under particular circumstances, such phenotypic plasticity can set the baseline for sympatric speciation and evolutionary divergence of habitat morphs (see Agrawal, 2001). In fact, environmental stress gradients in coastal intertidal habitats related to heat, desiccation, salinity, and wave action can provide the adequate setting for adaptive processes in patellids (Branch, 1981). If reproductive isolation is enforced by the ecological characteristics of each habitat, then biological separated species can be revealed. A good example comes from the diversification in *Nacella* limpets in the Magellanic Province (South America) (González‐Wevar, Nakano, Cañete, & Poulin, 2011). Ecological speciation and restricting levels of gene flow resulting from ecologically based divergent selection are considered the main driving processes of such diversification. The possibility of speciation along ecological gradients, without the need of a complete allopatric isolation, has also been shown for the Hawaiian endemic limpets of the genus *Cellana* (Bird, Holland, Bowen, & Toonen, 2011).

### 4.2 Population genetic structure and contemporary connectivity

Genetic differentiation estimates among archipelagos revealed a highly structured pattern in the *P. candei* complex across Macaronesia. Populations of *P. candei* from Azores are the most isolated and exhibit the highest level of differentiation from the remaining archipelagos. To a lesser extent, populations from Madeira and Canaries, which are about 400 km apart, also show significant genetic differentiation and limited contemporary connectivity. In fact, migratory events between Madeira and Canaries are unlikely, if not entirely absent, despite the fact that these archipelagos are geographically closer to each other. Only three individuals showed a very slightly probability of being migrants between these two archipelagos. Selvagens islands, which stand at approximately two‐thirds of the way between Madeira and the Canary Islands, and could act as a putative stepping stone for gene flow requires further examination. As for Azores, despite the wide geographical distribution of its islands across (~600 km), the minimum and maximum distances among any pair of adjacent islands that pelagic larvae must travel among islands are approximately 32 and 220 km, respectively. Such distances do not seem to offer an obstacle and allow populations’ gene pool across all islands to be homogenized via larval transport. Although genetic differentiation among archipelagos seems to be highly correlated with geographic distance, the unbalanced nature of sampling, the fact that connectivity within archipelago (i.e., Azores) does not follow IBD, and the results provided by BARRIER (see Figure 7) and BIMr analyses, suggest that the most likely barriers to gene flow in *P. candei* across the Macaronesian archipelagos are also associated to historical and contemporary limitations imposed by the masses of water that separate them, and are not a direct result of the geographical distance per se. In this case, the historical shifting of ocean circulation processes and the current oceanographic complexity and mesoscale variability across Macaronesia, with meanders, high eddy kinetic energy, upwellings, and several masses with distinct thermohaline characteristics (see Alves, Gaillard, Sparrow, Knoll, & Giraud, 2002; Johnson & Stevens, 2000; Rogerson, Rohling, Weaver, & Murray, 2004), may have acted as a strong physical barriers to gene exchange among archipelagos. Such barriers to gene flow, however, cannot fully exclude limitative dispersal across space or IBD pattern in *P. candei* throughout Macaronesia. Not only larval mortality rates increase almost exponentially as they move away from the coast into offshore waters (Cowen, Lwiza, Sponaugle, Paris, & Olson, 2000), but the PLD of *P. candei* may also be shorter than expected, or of a narrower range of what is generally referred for other patellids (Ribeiro, 2008). Under such scenario, because larvae are less likely to travel longer distances, populations farther away from each other would be more genetically distinct.

The failure of some microsatellites, which were isolated from the genome of *P. candei* samples from Azores, in amplifying individuals from Madeira and Canaries may further suggest substantial genetic break among archipelagos. The reduced marker polymorphism in southern samples may reflect pronounced sequence differences among subspecies due to their genetic divergence, thus entailing an upward ascertainment bias as markers were developed from Azorean morphotypes. This may explain the contrasting results of this study and those provided by Sá‐Pinto et al. (2005, 2008). In their study, samples from Azores and Madeira grouped together in a well‐supported clade, leaving Canaries more distant related. According to the same authors, the scenario of a single colonization event for each archipelago associated with the absence of historical gene flow between them is the most likely. The direction and timing of such colonization events is still unclear, and methods such as the approximate Bayesian computation (Beaumont, Zhang, & Balding, 2002) may be useful in contrasting demographic hypothesis about the evolutionary history of limpets in Macaronesia, provided that all archipelagos are sampled (including Selvagens). Even so, it seems plausible to accept that upon a single event of colonization, limpet populations in each archipelago remained isolated and evolved/adapted allopatrically to the environmental specificities of each archipelago. It is thought that *P. candei candei* from the Selvagens islands is the ancestral species that first colonized the Canaries and Madeira and only later the Azores (Sá‐Pinto et al., 2008; Weber & Hawkins, 2002). This evolutionary pattern tracks each archipelago's time of origin with Selvagens being the oldest (~29.5 Ma) and Azores the youngest (<6 Ma), but the exact sequence of colonization is still unresolved. Changes in sea level and ocean circulation associated with major historical episodes such as the tectonic closure of the Isthmus of Panama (Haug & Tiedemann, 1998), the Plio‐Pleistocene glacial cycles (Maggs et al., 2008), and the closing off of the western basin of the Mediterranean from the Atlantic (Krijgsman et al., 1999) may have contributed to the expansion and allopatric differentiation of *P. candei* throughout Macaronesia. As suggested for the endemic Macaronesia periwinkle *Tectarius striatus* (den Broeck, Breugelmans, De Wolf, & Backeljau, 2008), *P. candei* may have colonized Macaronesia in periods when sea levels were lower, so that seamounts peaked above sea level and acted as stepping stones between archipelagos. Therefore, adaptive processes associated with niche differentiation and physical/geographical isolation among populations may correspond to the underlying mechanisms for *P. candei* diversification in Macaronesia. In the absence of gene flow between populations, reproductive isolation would arise gradually as a result of mutation, genetic drift and natural selection driven by differences in local environmental conditions (Hoskins, Higgie, McDonald, & Moritz, 2005). In fact, allopatric speciation under restrictive gene flow is believed to be one of the most common modes of speciation in nature (Schluter, 2009). Yet, providing that reproductive isolation is complete, secondary events of colonization followed by the weakening of physical/ geographical barriers are not to be excluded and, for instance, may have contributed to the coexistence of *P. candei candei* (the Selvagens ecotype) and *P. candei crenata* in Canaries islands; although isolated specimens of *P. candei candei* were identified in El Hierro and Tenerife islands, this co‐occurrence is now mainly restricted to a single island: Fuerteventura (González‐Lorenzo et al., 2016).

### 4.3 Conservation of limpet population in Macaronesia

The aim of conservation is not simply to safeguard species from going extinct, but also to guarantee that morphological and genetic variation in natural populations is preserved. Efforts toward ensuring the conservation of limpet populations in Macaronesia are highly recommended, especially considering that their low genetic diversity and lack of gene exchange between archipelagos suggest they may be highly vulnerable. Taking into account its endemic nature and the negative impact of over‐exploitation in coastal communities, the risk of complete extinction of *P. candei* in Macaronesia is therefore conceivable. Yet, because our study failed to detect the occurrence of population bottlenecks, which would be expected under the known demographic decline of *P. candei* in Macaronesia, the effective population sizes must be still large enough to prevent critical losses to genetic variability (see Pujolar et al., 2011). However, when comparing to unexploited populations of patellids elsewhere (e.g., Perez et al., 2007; Ribeiro, Branco, Hawkins, & Santos, 2010), genetic diversity in the *P. candei* complex is fairly low. Despite the challenges associated with such comparisons, especially because different microsatellite loci may generate different levels of variation, the reduced genetic diversity observed can be a consequence of high levels of population inbreeding caused by intensive exploitation. As harvesting is mainly aimed at larger individuals (Martins et al., 2008), the more fertile individuals with higher reproductive outputs are likely less abundant than expected. This may lead to severe evolutionary and ecological consequences for the biology, life‐history traits, and survival of the species (Fenberg & Roy, 2008).

Our results support the view that populations from each archipelago should be managed for conservation as distinct units. Within the *P. candei* complex, *P. candei candei* is considered in danger of extinction under the Spanish Catalogue of Endangered Species and is now mainly restricted to one single island: Fuerteventura (Canary Islands). This is likely a consequence of overexploitation (Núñez, Brito, Riera, Docoito, & Monterroso, 2003), but selective and evolutionary‐related processes may have also been involved (González‐Lorenzo et al., 2016). Recently, a forced ban to its capture and a conservational plan has been put in place by regional authorities. Aside from Fuerteventura, *P. candei candei* occurs more abundantly in the Selvagens, uninhabitable islands that are protected under the Portuguese status of Nature Reserve. As for the remaining limpets, each Regional authority has established protective measures and rules for their harvesting. Legislation does not differ much among regions, with the establishment of limpet no‐take areas, minimum legal catch sizes, and seasonal fishing closures. Unfortunately, these actions have been largely ineffective in protecting such resource (Diogo, Pereira, & Schmiing, 2016; López et al., 2012; Martins et al., 2010; Riera et al., 2016), mostly because of illegal harvesting and lack of or insufficient enforcement. Protective measures need to be adjusted to the particular life‐history traits of *P. candei* in each archipelago (e.g., temporal variation in reproduction, recruitment, population dynamics). Furthermore, both environmental awareness programs to general population and coastal enforcement by local authorities should be stimulated. Above all, we suggest the establishment of fully enforced closed zones to limit the access to rocky shore poachers and allow limpet populations to grow in numbers and individual sizes. These off‐limit areas should be regularly surveyed and take into consideration population connectivity patterns within archipelagos. Our data suggest that, at least for Azores, such areas should distance no more than ~200 km, to allow proper gene exchange between populations. Additional sampling throughout all Macaronesia islands can further sharpen our knowledge about connectivity patterns in *P. candei* and help better defining the establishment of such areas within each archipelago.

Under the very probable assumption that *P. candei* from each archipelago forms a geographically and/or ecologically isolated population, the various subspecies within the *P. candei* complex as previously proposed by Christiaens (1973) may be best thought as true species using the denomination: *Patella candei* in Selvagens, *Patella gomesii* in Azores, *Patella ordinaria* in Madeira, and *Patella crenata* for Canaries. Whether this would facilitate current taxonomic misinterpretations and conservation needs, further research is still required, especially in diagnosing intrinsic reproductive isolation (Frankham et al., 2012). Ultimately, this elevation of subspecies to species level would be in agreement with stock delimitation and units of conservation (Hawkins et al., 2016) with potential benefits for management plans aimed at the preservation of limpets stocks across the Macaronesia region.

## ACKNOWLEDGMENTS

We sincerely thank two anonymous reviewers for their useful comments that improved significantly this manuscript. We thank Manuel Rivas and Manuel Enes for helping with DNA extractions and morphometric measurements, respectively. Field and sampling assistance was provided by Maria Vale, Afonso Prestes, Joana Pombo in Santa Maria (Azores), José Azevedo in Pico (Azores), André Amaral in Terceira (Azores), Pedro Raposeiro in Flores and Corvo (Azores), and Fernando Tuya and Manuel Rivas in Canarias. We thank Michael Collyer (Western Kentucky University) for help in geometric morphometrics. This research was partially supported by the European Regional Development Fund (ERDF) through the COMPETE—Operational Competitiveness Programme and national funds through FCT—Foundation for Science and Technology, under the projects PTDC/BIA‐BIC/115837/2009 and PEst‐C/MAR/LA0015/2013, by the Strategic Funding UID/Multi/04423/2013 through national funds provided by FCT—Foundation for Science and Technology and European Regional Development Fund (ERDF), in the framework of the programme PT2020 and by cE3c funding (Ref: UID/BIA/00329/2013). JF was funded by a PhD grant M3.1.2/ F/021/2011 by the Regional Government of the Azores. GMM was supported by postdoctoral grants awarded by FCT, Portugal (SFRH/ BDP/63040/2009). PAR was funded by the Portuguese Foundation for Science and Technology, through a postdoctoral grant ref. SFRH/BPD/69232/2010 funded through QREN and COMPETE, and the strategic project UID/MAR/04292/2013 granted to MARE.

## CONFLICT OF INTEREST

None declared.

## DATA ACCESSIBILITY

Data used for this article (geographic distances, morphometric data, microsatellite genotypes, R scripts) have been deposited in Dryad, https://doi.org/datadryad.org/review?doi=10.5061/dryad.d3vk4.

## AUTHOR CONTRIBUTIONS

JF and GMM conceived the study and all co‐authors equally contributed to its final design. JF performed all the analyses. AP and PP assisted JF with the validation of genotypes and genetic analyses. GMM assisted JF with multivariate analysis. AIN coordinated sampling. JF and PR were involved in sample collection. JF interpreted the results with input from GMM, SJH, PP, and AIN. JF wrote the manuscript with input from all co‐authors.

## References

[ece33121-bib-0001] Adams, D. C. , Collyer, M. , Kaliontzopoulou, A. , & Sherratt, E. (2016). geomorph: Geometric morphometric analyses of 2D/3D landmark data 3.0.2. Available from: URL https://cran.r-project.org/package=geomorph

[ece33121-bib-0002] Adams, D. C. , & Otarola‐Castillo, E. (2013). geomorph: An R package for the collection and analysis of geometric morphometric shape data. Methods in Ecology and Evolution, 4, 393–399.

[ece33121-bib-0003] Adams, D. C. , Rohlf, F. J. , & Slice, D. E. (2013). A field comes of age: Geometric morphometrics in the 21st century. Hystrix, the Italian Journal of Mammalogy, 24, 7–14.

[ece33121-bib-0004] Agrawal, A. A. (2001). Phenotypic plasticity in the interactions and evolution of species. Science, 294, 321–326. 11598291 10.1126/science.1060701

[ece33121-bib-0005] Alves, M. , Gaillard, F. , Sparrow, M. , Knoll, M. , & Giraud, S. (2002). Circulation patterns and transport of the Azores Front‐Current system. Deep Sea Research Part II: Topical Studies in Oceanography, 49, 3983–4002.

[ece33121-bib-0006] Anderson, M. J. (2001). A new method for non‐parametric multivariate analysis of variance. Austral Ecology, 26, 32–46.

[ece33121-bib-0007] Anderson, M. J. , Gorley, R. N. , & Clarke, K. R. (2008) Permanova+ for Primer: Guide to software and statistical methods. Plymouth, UK: PRIMER‐E.

[ece33121-bib-0008] Ávila, S. , Melo, C. , Berning, B. , Cordeiro, R. , Landau, B. , & da Silva, C. M. (2016). *Persististrombus coronatus* (Mollusca: Strombidae) in the lower Pliocene of Santa Maria Island (Azores, NE Atlantic): Paleoecology, paleoclimatology and paleobiogeographic implications. Palaeogeography, Palaeoclimatology, Palaeoecology, 441, 912–923.

[ece33121-bib-0009] Barber, P. H. , Palumbi, S. R. , Erdmann, M. V. , & Moosa, M. K. (2000). A marine Wallace's line? Nature, 406, 692–693. 10963585 10.1038/35021135

[ece33121-bib-0010] Baylac, M. , Villemant, C. , & Simbolotti, G. (2003). Combining geometric morphometrics with pattern recognition for the investigation of species complexes. Biological Journal of the Linnean Society, 80, 89–98.

[ece33121-bib-0011] Beaumont, M. A. , Zhang, W. , & Balding, D. J. (2002). Approximate Bayesian computation in population genetics. Genetics, 162, 2025–2035. 12524368 10.1093/genetics/162.4.2025 PMC1462356

[ece33121-bib-0012] Bird, C. E. , Holland, B. S. , Bowen, B. W. , & Toonen, R. J. (2011). Diversification of sympatric broadcast‐spawning limpets (*Cellana* spp.) within the Hawaiian archipelago. Molecular Ecology, 20, 2128–2141. 21481050 10.1111/j.1365-294X.2011.05081.x

[ece33121-bib-0013] Boaventura, D. , Alexander, M. , Santina, P. D. , Smith, N. D. , Ré, P. , da Fonseca, L. C. , & Hawkins, S. J. (2002). The effects of grazing on the distribution and composition of low‐shore algal communities on the central coast of Portugal and on the southern coast of Britain. Journal of Experimental Marine Biology and Ecology, 267, 185–206.

[ece33121-bib-0014] Bookstein, F. L. (1991). Morphometric tools for landmark data: Geometry and biology. New York, NY: Cambridge University Press.

[ece33121-bib-0015] Branch, G. M. (1981). The biology of limpets: Physical factors, energy flow, and ecological interactions. Oceanography and Marine Biology: An Annual Review, 19, 235–380.

[ece33121-bib-0016] Branch, G. M. , & Marsh, A. C. (1978). Tenacity and shell shape in six *Patella* species: Adaptive features. Journal of Experimental Marine Biology and Ecology, 3, 111–130.

[ece33121-bib-0017] Cabral, J. P. (2007). Shape and growth in European Atlantic *Patella* limpets (Gastropoda, Mollusca). Ecological implications for survival. Web Ecology, 7, 11–21.

[ece33121-bib-0018] Carlsson, J. (2008). Effects of microsatellite null alleles on assignment testing. Journal of Heredity, 99, 616–623. 18535000 10.1093/jhered/esn048

[ece33121-bib-0019] Chapuis, M. P. , & Estoup, A. (2007). Microsatellite null alleles and estimation of population differentiation. Molecular Biology and Evolution, 24, 621–631. 17150975 10.1093/molbev/msl191

[ece33121-bib-0020] Christiaens, J. (1973). Révision du genre *Patella* (Mollusca, Gastropoda). Bulletin du Muséum National D'histoire Naturelle, 182, 1305–1392.

[ece33121-bib-0021] Coleman, R. A. , Underwood, A. J. , Benedetti‐Cecchi, Aberg. P. , Arenas, F. , Arrontes, J. , Castro, J. , … Hawkins, S. J. (2006). A continental scale evaluation of the role of limpet grazing on rocky shores. Oecologia, 147, 556–564. 16450182 10.1007/s00442-005-0296-9

[ece33121-bib-0022] Collyer, M. L. , Sekora, D. J. , & Adams, D. C. (2015). A method for analysis of phenotypic change for phenotypes described by high‐dimensional data. Heredity, 115, 357–365. 25204302 10.1038/hdy.2014.75 PMC4815463

[ece33121-bib-0023] Côrte‐Real, H. B. S. M. , Hawkins, S. J. , & Thorpe, J. P. (1996). Population differentiation and taxonomic status of the exploited limpet *Patella candei* in the Macaronesian Islands (Azores, Madeira, Canaries). Marine Biology, 125, 141–152.

[ece33121-bib-0024] Cowen, R. K. , Lwiza, K. M. M. , Sponaugle, S. , Paris, C. B. , & Olson, D. B. (2000). Connectivity of marine populations: Open or closed? Science, 287, 857–859. 10657300 10.1126/science.287.5454.857

[ece33121-bib-0025] Cowen, R. K. , & Sponaugle, S. (2009). Larval dispersal and marine population connectivity. Annual Review of Marine Science, 1, 443–466. 10.1146/annurev.marine.010908.163757 21141044

[ece33121-bib-0026] Davis, M. A. , Douglas, M. R. , Collyer, M. L. , & Douglas, M. E. (2016). Deconstructing a species‐complex: Geometric morphometric and molecular analyses define species in the western rattlesnake (*Crotalus viridis*). PLoS ONE, 11, e0146166. 26816132 10.1371/journal.pone.0146166 PMC4731396

[ece33121-bib-0027] De Wolf, H. , Backeljau, T. , Medeiros, R. , & Verhagen, R. (1997). Microgeographical shell variation in *Littorina striata*, a planktonic developing periwinkle. Marine Biology, 129, 331–342.

[ece33121-bib-0028] den Broeck, H. V. , Breugelmans, K. , De Wolf, H. , & Backeljau, T. (2008). Completely disjunct mitochondrial DNA haplotype distribution without a phylogeographic break in a planktonic developing gastropod. Marine Biology, 153, 421–429.

[ece33121-bib-0029] Denny, M. W. (2000). Limits to optimization: Fluid dynamics, adhesive strength and the evolution of shape in limpet shells. Journal of Experimental Biology, 203, 2603–2622. 10934003 10.1242/jeb.203.17.2603

[ece33121-bib-0030] Diogo, H. , Pereira, J. G. , & Schmiing, M. (2016). Catch me if you can: Non‐compliance of limpet protection in the Azores. Marine Policy, 63, 92–99.

[ece33121-bib-0031] Dodd, J. M. (1957). Artificial fertilisation, larval development and metamorphosis in *Patella vulgata* L. & *Patella caerulea* L. Pubblicazioni della Stazione Zoologica di Napoli, 29, 172–185.

[ece33121-bib-0032] Earl, D. A. , & vonHoldt, B. M. (2012). STRUCTURE HARVESTER: A website and program for visualizing STRUCTURE output and implementing the Evanno method. Conservation Genetics Resources, 4, 359–361.

[ece33121-bib-0033] Evanno, G. , Regnaut, S. , & Goudet, J. (2005). Detecting the number of clusters of individuals using the software STRUCTURE: A simulation study. Molecular Ecology, 14, 2611–2620. 15969739 10.1111/j.1365-294X.2005.02553.x

[ece33121-bib-0034] Excoffier, L. , & Lischer, H. E. L. (2010). Arlequin suite ver 3.5: A new series of programs to perform population genetics analyses under Linux and Windows. Molecular Ecology Resources, 10, 564–567. 21565059 10.1111/j.1755-0998.2010.02847.x

[ece33121-bib-0035] Faria, J. , Froufe, E. , Tuya, F. , Alexandrino, P. , & Pérez‐Losada, M. (2013). Panmixia in the endangered slipper lobster *Scyllarides latus* from the Northeastern Atlantic and Western Mediterranean. Journal of Crustacean Biology, 33, 557–566.

[ece33121-bib-0036] Faria, J. , Pita, A. , Rivas, M. , Martins, G. M. , Hawkins, S. J. , Ribeiro, P. , … Presa, P. (2016). A multiplex microsatellite tool for conservation genetics of the endemic limpet *Patella candei* in the Macaronesian archipelagos. Aquatic Conservation: Marine and Freshwater Ecosystems, 26, 775–781.

[ece33121-bib-0037] Faubet, P. , & Gaggiotti, O. E. (2008). A new Bayesian method to identify the environmental factors that influence recent migration. Genetics, 178, 1491–1504. 18245344 10.1534/genetics.107.082560 PMC2278086

[ece33121-bib-0038] Faubet, P. , Waples, R. S. , & Gaggiotti, O. E. (2007). Evaluating the performance of a multilocus Bayesian method for the estimation of migration rates. Molecular Ecology, 16, 1149–1166. 17391403 10.1111/j.1365-294X.2007.03218.x

[ece33121-bib-0039] Fenberg, P. B. , & Roy, K. (2008). Ecological and evolutionary consequences of size‐selective harvesting: How much do we know? Molecular Ecology, 17, 209–220. 17868288 10.1111/j.1365-294X.2007.03522.x

[ece33121-bib-0040] Frankham, R. , Ballou, J. D. , Dudash, M. R. , Eldrigde, M. D. B. , Fenster, C. B. , Lacy, R. C. , … Ryder, O. A. (2012). Implications of different species concepts for conserving biodiversity. Biological Conservation, 153, 25–31.

[ece33121-bib-0041] Gerlach, G. , Jueterbock, A. , Kraemer, P. , Deppermann, J. , & Harmand, P. (2010). Calculations of population differentiation based on G_(ST)_ and D: Forget G_(ST)_ but not all of statistics!. Molecular Ecology, 19, 3845–3852. 20735737 10.1111/j.1365-294X.2010.04784.x

[ece33121-bib-0042] González‐Lorenzo, G. , Hernández, E. M. , Pérez‐Dionis, G. , Hernández, A. B. , Santos, B. G. , & Diez, J. B. (2016). Ineffective conservation threatens *Patella candei*, an endangered limpet endemic to the Macaronesian islands. Biological Conservation, 192, 428–435.

[ece33121-bib-0043] González‐Wevar, C. A. , Nakano, T. , Cañete, J. I. , & Poulin, E. (2011). Concerted genetic, morphological and ecological diversification in Nacella limpets in the Magellanic Province. Molecular Ecology, 20, 1936–1951. 21418364 10.1111/j.1365-294X.2011.05065.x

[ece33121-bib-0044] Goudet, J. (1995). FSTAT (version 1.2): A computer program to calculate F‐statistics. Journal of Heredity, 86, 485–486.

[ece33121-bib-0045] Guerra‐Varela, J. , Colson, I. , Backeljau, T. , Breugelmans, K. , Hughes, R. N. , & Rolán‐Alvarez, E. (2009). The evolutionary mechanism maintaining shell shape and molecular differentiation between two ecotypes of the dogwhelk *Nucella lapillus* . Evolutionary Ecology, 23, 261–280.

[ece33121-bib-0046] Guichoux, E. , Lagache, L. , Wagner, S. , Chaumeil, P. , Leger, P. , Lepais, O. , … Petit, R. J. (2011). Current trends in microsatellite genotyping. Molecular Ecology Resources, 11, 591–611. 21565126 10.1111/j.1755-0998.2011.03014.x

[ece33121-bib-0047] Gunz, P. , & Mitteroecker, P. (2013). Semilandmarks: A method for quantifying curves and surfaces. Hystrix, the Italian Journal of Mammalogy, 24, 103–109.

[ece33121-bib-0048] Harley, C. D. G. , Denny, M. W. , Mach, K. J. , & Miller, L. P. (2009). Thermal stress and morphological adaptations in limpets. Functional Ecology, 23, 292–301.

[ece33121-bib-0049] Haug, G. H. , & Tiedemann, R. (1998). Effect of the formation of the Isthmus of Panama on Atlantic Ocean thermohaline circulation. Nature, 393, 673–678.

[ece33121-bib-0050] Hawkins, S. J. , Bohn, K. , Sims, D. W. , Ribeiro, P. , Faria, J. , Presa, P. , Pita, A. , Martins, G. M. , Neto, A. I. , Burrows, M. T. & Genner, M. J. (2016). Fisheries stocks from an ecological perspective: Disentangling ecological connectivity from genetic interchange. Fisheries Research, 179, 333–341.

[ece33121-bib-0051] Hawkins, S. J. , Côrte‐Real, H. B. S. M. , Martins, H. R. , Santos, R. S. , & Martins, A. M. F. (1990). A note on the identity of *Patella* in the Azores. Açoreana (Suppl.), 167–173.

[ece33121-bib-0052] Hawkins, S. J. , Côrte‐Real, H. B. S. M. , Pannacciulli, F. G. , Weber, L. C. , & Bishop, J. D. D. (2000). Thoughts on the ecology and evolution of the intertidal biota of the Azores and other Atlantic islands. Hydrobiologia, 440, 3–17.

[ece33121-bib-0053] Hawkins, S. J. , & Hartnoll, R. G. (1983). Grazing of intertidal algae by marine invertebrates. Oceanography and Marine Biology: An Annual Review, 21, 195–282.

[ece33121-bib-0054] Hoskins, C. J. , Higgie, M. , McDonald, K. R. , & Moritz, C. (2005). Reinforcement drives rapid allopatric speciation. Nature, 437, 1353–1356. 16251964 10.1038/nature04004

[ece33121-bib-0055] Johnson, J. , & Stevens, I. (2000). A fine resolution model of the eastern North Atlantic between the Azores, the Canary Islands and the Gibraltar Strait. Deep Sea Research Part I: Oceanographic Research Papers, 47, 875–899.

[ece33121-bib-0056] Jombart, T. (2008). adegenet: A R package for the multivariate analysis of genetic markers. Bioinformatics, 24, 1403–1405. 18397895 10.1093/bioinformatics/btn129

[ece33121-bib-0057] Jombart, T. , Devillard, S. , & Balloux, F. (2010). Discriminant analysis of principal components: A new method for the analysis of genetically structured populations. BMC Genetics, 11, 94. 20950446 10.1186/1471-2156-11-94 PMC2973851

[ece33121-bib-0058] Jost, L. (2008). G_ST_ and its relatives do not measure differentiation. Molecular Ecology, 17, 4015–4026. 19238703 10.1111/j.1365-294x.2008.03887.x

[ece33121-bib-0059] Keever, C. C. , Sunday, J. , Puritz, J. B. , Addison, J. A. , Toonen, R. J. , Grosberg, R. K. , & Hart, M. W. (2009). Discordant distribution of populations and genetic variation in a sea star with high dispersal potential. Evolution, 63, 3214–3227. 19663996 10.1111/j.1558-5646.2009.00801.x

[ece33121-bib-0060] Kelly, R. P. , & Palumbi, S. R. (2010). Genetic structure among 50 species of the Northeastern Pacific rocky intertidal community. PLoS ONE, 5, e8594. 20062807 10.1371/journal.pone.0008594 PMC2799524

[ece33121-bib-0061] Krijgsman, W. , Hilgen, F. J. , Raffi, I. , Sierra, F. J. , & Wilson, D. S. (1999). Chronology, causes and progression of the Messinian salinity crisis. Nature, 400, 652–655.

[ece33121-bib-0062] Lande, R. (1988). Genetics and demography in biological conservation. Science, 241, 1455–1460. 3420403 10.1126/science.3420403

[ece33121-bib-0063] López, C. , Poladura, A. , Hernández, J. C. , Martín, L. , Conceptíon, L. , Sangil, C. , & Clemente, S. (2012). Contrasting effects of protection from harvesting in populations of two limpet species in a recently established marine protected area. Scientia Marina, 76, 799–807.

[ece33121-bib-0064] Lowell, R. B. (1986). Crab predation on limpets: Predator behavior and defensive features of the shell morphology of the prey. Biological Bulletin, 171, 577–596. 10.2307/1541625 29314885

[ece33121-bib-0065] Maggs, C. A. , Castilho, R. , Foltz, D. , Henzler, C. , Jolly, M. T. , Kelly, J. , … Wares, J. (2008). Evaluating signatures of the glacial refugia for North Atlantic benthic marine taxa. Ecology, 89, 108–122. 10.1890/08-0257.1 19097488

[ece33121-bib-0501] Manni, F. , Guérard, E. , Heyer, E. (2004). Geographic patterns of (genetic, morphologic, linguistic) variation: how barriers can be detected by using Monmonier’s algorithm. Human Biology, 76, 173–190. 15359530 10.1353/hub.2004.0034

[ece33121-bib-0066] Martins, G. M. , Borges, C. D. G. , Vale, M. , Ferraz, R. R. , Martins, H. R. , Santos, R. S. , & Hawkins, S. J. (2017). Exploitation promotes earlier sex change in a protandrous patellid limpet, *Patella aspera* Röding, 1798. Ecology and Evolution, 7, 3616–3622. 28515897 10.1002/ece3.2925 PMC5433971

[ece33121-bib-0067] Martins, G. M. , Jenkins, S. R. , Hawkins, S. J. , Neto, A. I. , Medeiros, A. R. , & Thompson, R. C. (2010). Illegal harvesting affects the success of fishing closure areas. Journal of the Marine Biological Association of the United Kingdom, 91, 929–937.

[ece33121-bib-0068] Martins, G. M. , Jenkins, S. R. , Hawkins, S. J. , Neto, A. I. , & Thompson, R. C. (2008). Exploitation of rocky intertidal grazers: Population status and potential impacts on community structure and functioning. Aquatic Biology, 3, 1–10.

[ece33121-bib-0069] Martins, H. R. , Santos, R. S. , & Hawkins, S. J. (1987). Exploitation of limpets (*Patella* spp.) in the Azores with a preliminary analysis of the stocks. ICES Report 1987/K, 53, 1–17.

[ece33121-bib-0070] Martins, G. M. , Thompson, R. C. , Neto, A. I. , Hawkins, S. J. , & Jenkins, S. R. (2010). Exploitation of intertidal grazers as a driver of community divergence. Journal of Applied Ecology, 47, 1282–1289.

[ece33121-bib-0071] McKay, J. K. , & Latta, R. G. (2002). Adaptive population divergence: Markers, QTL and traits. Trends in Ecology and Evolution, 17, 285–291.

[ece33121-bib-0072] Meirmans, P. G. (2014). Non‐convergence in Bayesian estimation of migration rates. Molecular Ecology Resources, 14, 726–733. 24373147 10.1111/1755-0998.12216

[ece33121-bib-0073] Núñez, J. , Brito, M. C. , Riera, R. , Docoito, J. R. , & Monterroso, O. (2003). Distribución actual de las poblaciones de Patella candei D'Orbigny, 1840 (Mollusca, Gastropoda) en las islas Canarias. Una especie en peligro de extinción. Boletin Instituto Espanol de Oceanografia, 19, 371–377.

[ece33121-bib-0074] Peakall, R. , & Smouse, P. E. (2006). GENALEX 6: Genetic analysis in Excel. Population genetic software for teaching and research. Molecular Ecology Notes, 6, 288–295. 10.1093/bioinformatics/bts460 PMC3463245 22820204

[ece33121-bib-0075] Perez, M. , Branco, M. , Llavona, A. , Ribeiro, P. A. , Santos, A. M. , Hawkins, S. J. , … Alexandrino, P. (2007). Development of microsatellite loci for the black‐footed limpet, *Patella depressa*, and cross‐amplification in two other *Patella* species. Conservation Genetics, 8, 739–742.

[ece33121-bib-0076] Portnoy, D. S. , Hollenbeck, C. M. , Belcher, C. N. , Driggers, W. B. III , Frazier, B. S. , Gelsleichter, J. , … Gold, J. R. (2014). Contemporary population structure and post‐glacial genetic demography in a migratory marine species, the blacknose shark, *Carcharhinus acronotus* . Molecular Ecology, 23, 5480–5495. 25294029 10.1111/mec.12954

[ece33121-bib-0077] Pritchard, J. K. , Stephens, M. , & Donnelly, P. (2000). Inference of population structure using multilocus genotype data. Genetics, 155, 945–959. 10835412 10.1093/genetics/155.2.945 PMC1461096

[ece33121-bib-0078] Pujolar, J. M. , Bevacqua, D. , Capoccioni, F. , Ciccoti, E. , De Leo, G. A. , & Zane, L. (2011). No apparent genetic bottleneck in the demographically declining European eel using molecular genetics and forward‐time simulations. Conservation Genetics, 12, 813–825.

[ece33121-bib-0079] R Core Team (2014). R: A language and environment for statistical computing. Vienna, Austria: R Foundation for Statistical Computing URL http://www.R-project.org/

[ece33121-bib-0080] Raymond, M. , & Rousset, F. (1995). GENEPOP (version 1.2)—population genetics software for exact tests and ecumenicism. Journal of Heredity, 86, 248–249.

[ece33121-bib-0081] Ribeiro, P. A. (2008) Dispersal and connectivity of northeastern Atlantic patellid limpets: A multidisciplinary approach. PhD thesis, UK: University of Southampton.

[ece33121-bib-0082] Ribeiro, P. A. , Branco, M. , Hawkins, S. J. , & Santos, A. M. (2010). Recent changes in the distribution of a marine gastropod, *Patella rustica*, across the Iberian Atlantic coast did not result in diminished genetic diversity or increased connectivity. Journal of Biogeography, 37, 1782–1796.

[ece33121-bib-0083] Riera, R. , Pérez, Ó. , Álvarez, O. , Simón, D. , Díaz, D. , & Monterroso, Núnez. J. (2016). Clear regression of harvested intertidal mollusks. A 20‐year (1994–2014) comparative study. Marine Environmental Research, 113, 56–61. 26606106 10.1016/j.marenvres.2015.11.003

[ece33121-bib-0084] Rogerson, M. , Rohling, E. G. , Weaver, P. P. E. , & Murray, J. W. (2004). The Azores front since the last glacial maximum. Earth and Planetary Science Letters, 222, 779–789.

[ece33121-bib-0085] Rohlf, F. J. (2015). The TPS series of software. Hystrix, the Italian Journal of Mammalogy, 26, 1–4.

[ece33121-bib-0086] Rohlf, F. J. , & Slice, D. (1990). Extensions of the Procrustes method for the optimal superimposition of landmarks. Systematic Zoology, 39, 40–59.

[ece33121-bib-0087] Rousset, F. (1997). Genetic differentiation and estimation of gene flow from F‐statistics under isolation by distance. Genetics, 145, 1219–1228. 9093870 10.1093/genetics/145.4.1219 PMC1207888

[ece33121-bib-0088] Ruane, S. (2015). Using geometric morphometrics for integrative taxonomy: An examination of head shapes of milksnakes (genus *Lampropeltis*). Zoological Journal of the Linnean Society, 174, 394–413.

[ece33121-bib-0089] Sandoval‐Castillo, J. , & Beheregaray, L. B. (2015). Metapopulation structure informs conservation management in a heavily exploited coastal shark (*Mustelus henlei*). Marine Ecology Progress Series, 533, 191–203.

[ece33121-bib-0090] Santos, R. S. , Hawkins, S. J. , Monteiro, L. R. , Alves, M. , & Isidro, E. J. (1995). Marine research, resources and conservation in the Azores. Aquatic Conservation: Marine and Freshwater Ecosystems, 5, 311–354.

[ece33121-bib-0091] Sá‐Pinto, A. , Branco, M. S. , Harris, D. J. , & Alexandrino, P. (2005). Phylogeny and phylogeography of the genus *Patella* based on mitochondrial DNA sequence data. Journal of Experimental Marine Biology and Ecology, 325, 95–110.

[ece33121-bib-0092] Sá‐Pinto, A. , Branco, A. , Sayanda, D. , & Alexandrino, P. (2008). Patterns of colonization, evolution and gene flow in species of the genus *Patella* in the Macaronesian Islands. Molecular Ecology, 17, 519–532. 18179442 10.1111/j.1365-294X.2007.03563.x

[ece33121-bib-0093] Schluter, D. (2009). Evidence for ecological speciation and its alternative. Science, 323, 737–741. 19197053 10.1126/science.1160006

[ece33121-bib-0094] Sokolova, I. M. , & Berger, V. J. (2000). Physiological variation related to shell colour polymorphism in White Sea *Littorina saxatilis* . Journal of Experimental Marine Biology and Ecology, 245, 1–23.

[ece33121-bib-0095] Szpiech, Z. A. , Jakobsson, M. , & Rosenberg, N. A. (2008). ADZE: A rarefaction approach for counting alleles private to combinations of populations. Bioinformatics, 27, 2498–2504. 10.1093/bioinformatics/btn478 PMC2732282 18779233

[ece33121-bib-0096] Trussell, G. C. (2000). Phenotypic clines, plasticity, and morphological trade‐offs in an intertidal snail. Evolution, 54, 151–166. 10937192 10.1111/j.0014-3820.2000.tb00016.x

[ece33121-bib-0097] Van Oosterhout, C. , Hutchinson, W. F. , Wills, D. P. M. , & Shipley, P. (2004). Micro‐checker: Software for identifying and correcting genotyping errors in microsatellite data. Molecular Ecology Notes, 4, 535–538.

[ece33121-bib-0098] Verhoeven, K. J. F. , Simonsen, K. L. , & Mcintyre, L. M. (2005). Implementing false discovery rate control: Increasing your power. Oikos, 3, 643–647.

[ece33121-bib-0099] Villegas, J. , Feliciangeli, M. D. , & Dujardin, J. P. (2002). Wing shape divergence between *Rhodnius prolixus* from Cojedes (Venezuela) and *R. robustus* from Mérida (Venezuela). Infection, Genetics and Evolution, 2, 121–128. 10.1016/s1567-1348(02)00095-3 12797988

[ece33121-bib-0100] Weber, L. I. , & Hawkins, S. J. (2002). Evolution of the limpet *Patella candei* d'Orbigny (Mollusca: Patellidae) in Atlantic archipelagos: Human intervention and natural processes. Biological Journal of the Linnean Society, 77, 341–353.

[ece33121-bib-0101] White, T. A. , Fotherby, H. A. , Stephens, P. A. , & Hoelzel, A. R. (2011). Genetic panmixia and demographic dependence across the North Atlantic in the deep‐sea fish, blue hake (*Antimora rostrata*). Heredity, 106, 690–699. 20717157 10.1038/hdy.2010.108 PMC3183912

[ece33121-bib-0102] Wilson, G. A. , & Rannala, B. (2003). Bayesian inference of recent migration rates using multilocus genotypes. Genetics, 163, 1177–1191. 12663554 10.1093/genetics/163.3.1177 PMC1462502

[ece33121-bib-0103] Wright, S. (1943). Isolation by distance. Genetics, 28, 114–138. 17247074 10.1093/genetics/28.2.114 PMC1209196

[ece33121-bib-0104] Zelditch, M. L. , Swiderski, D. L. , & Sheets, H. D. (2012). Geometric morphometrics for biologists: A primer, 2nd ed London, UK: Elsevier Academic Press. 478 pp.

