# Supplementary material for: Disentangling the genetic and morphological structure of Patella candei complex in Macaronesia (NE Atlantic)
Source: Ecol Evol. 2017 Jun 29;7(16):6125–40. doi: 10.1002/ece3.3121 (PMC5574786; doi:10.1002/ece3.3121)
Supplement: Supplementary file 1 [file ECE3-7-6125-s001.docx]

**Supporting information**

**
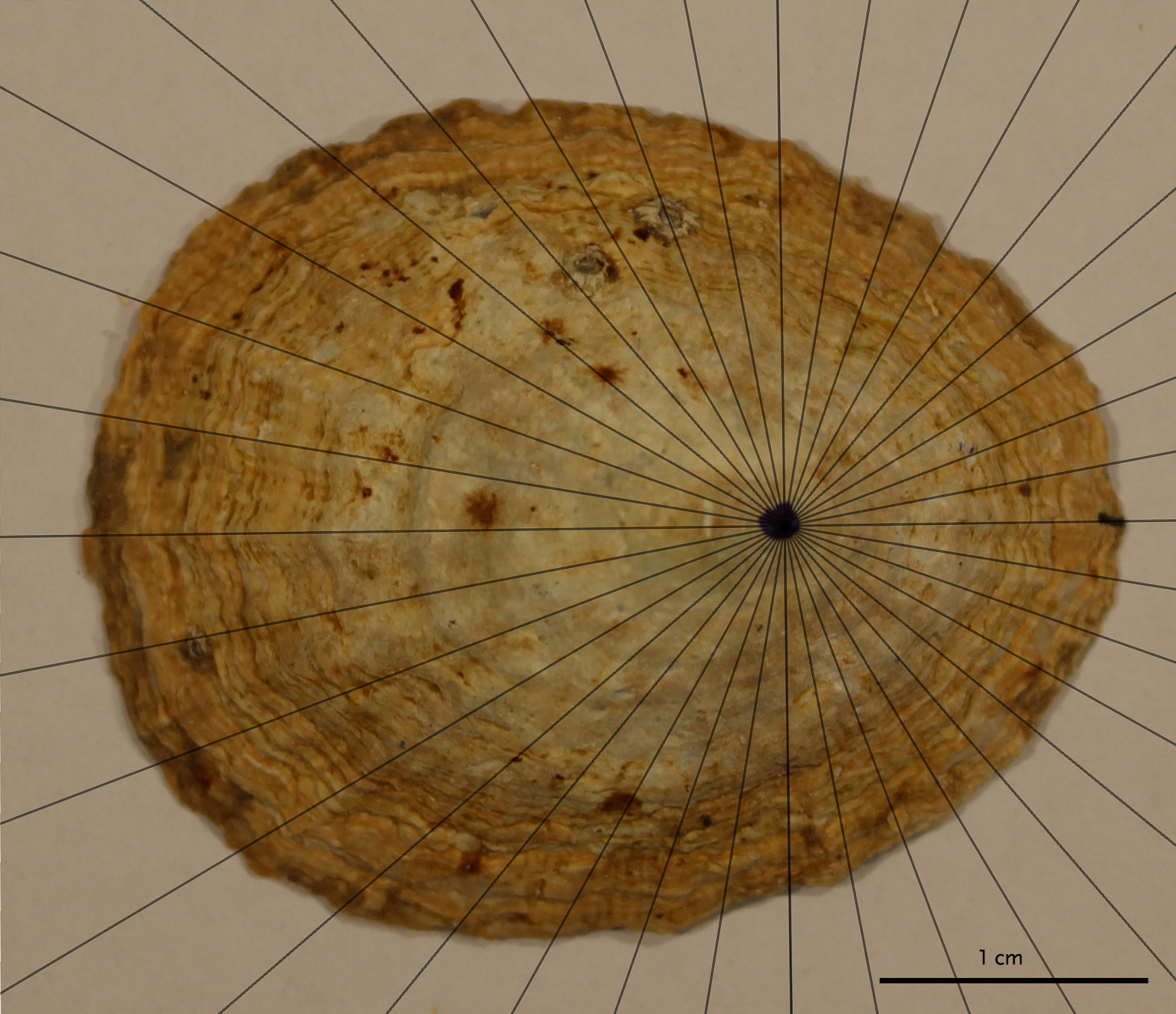
**

**Figure S1.** Representative imaging of a *Patella candei* shell used for geometric morphometrics. Shells were oriented on a fan by superimposed the anterior and posterior ends along the horizontal line of the fan. The apex was made to coincide with the vertical line of the fan and all shells were placed in the fan with their anterior end facing the right side. The fan consisted of a set of 18 lines. angularly distanced by 10º increments.


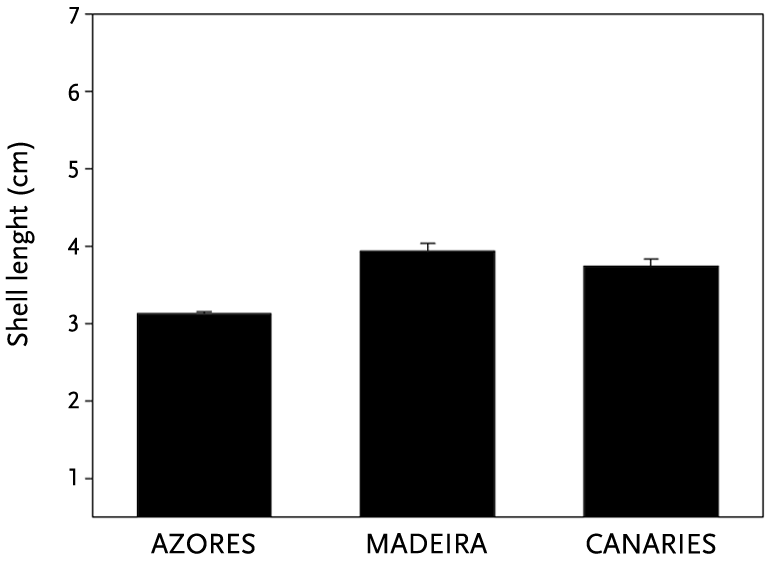


**Figure S2**. Mean (+SE) *Patella candei* shell length across archipelagos.

**Figure S3**. Regression analysis of ontogenetic trajectories for each archipelago against log(CS) using the regression score as shape variable.

**
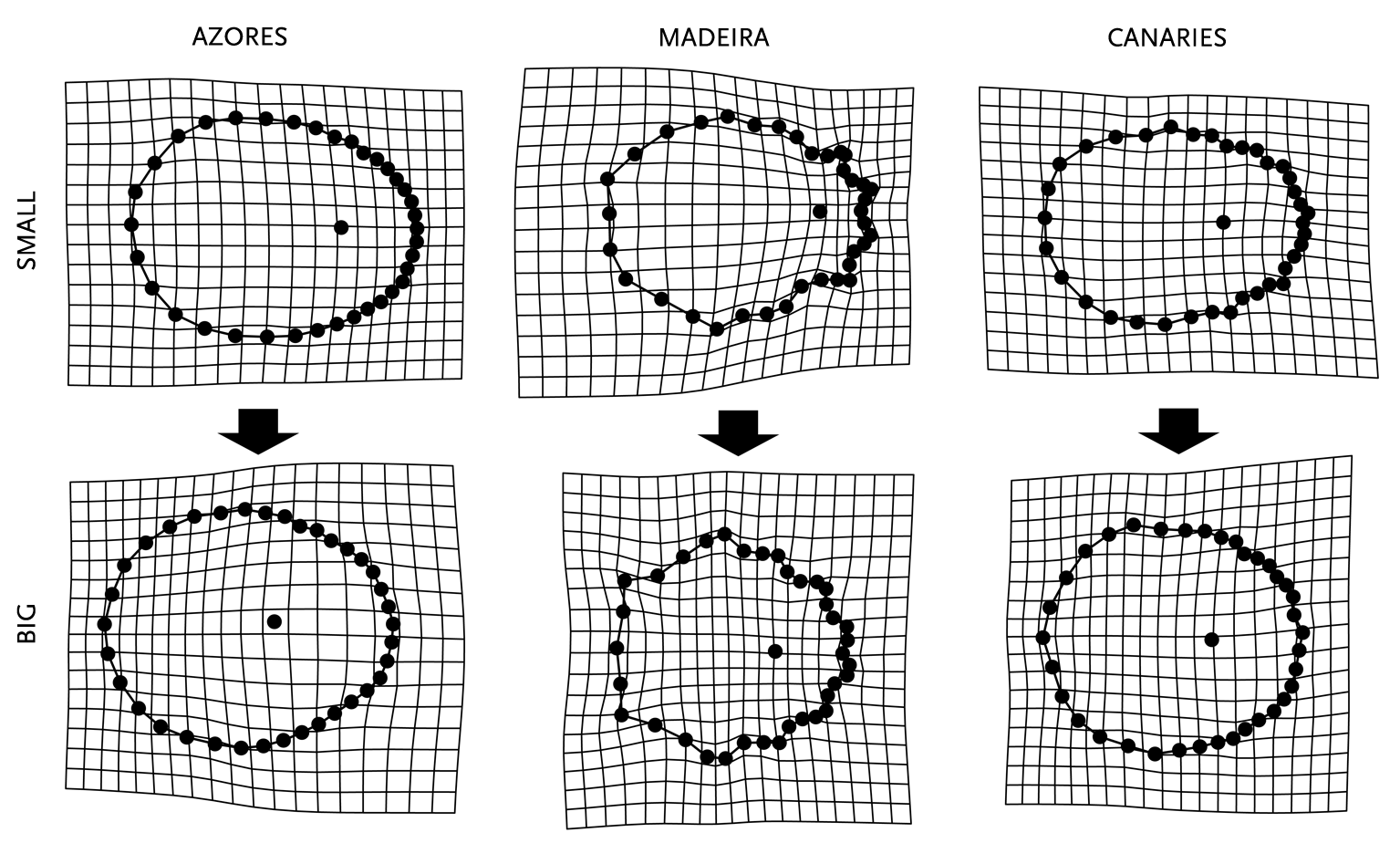
**

**Figure S4**. Deformation grids for shell shape variation of the three smaller and bigger limpets of each archipelago.

**
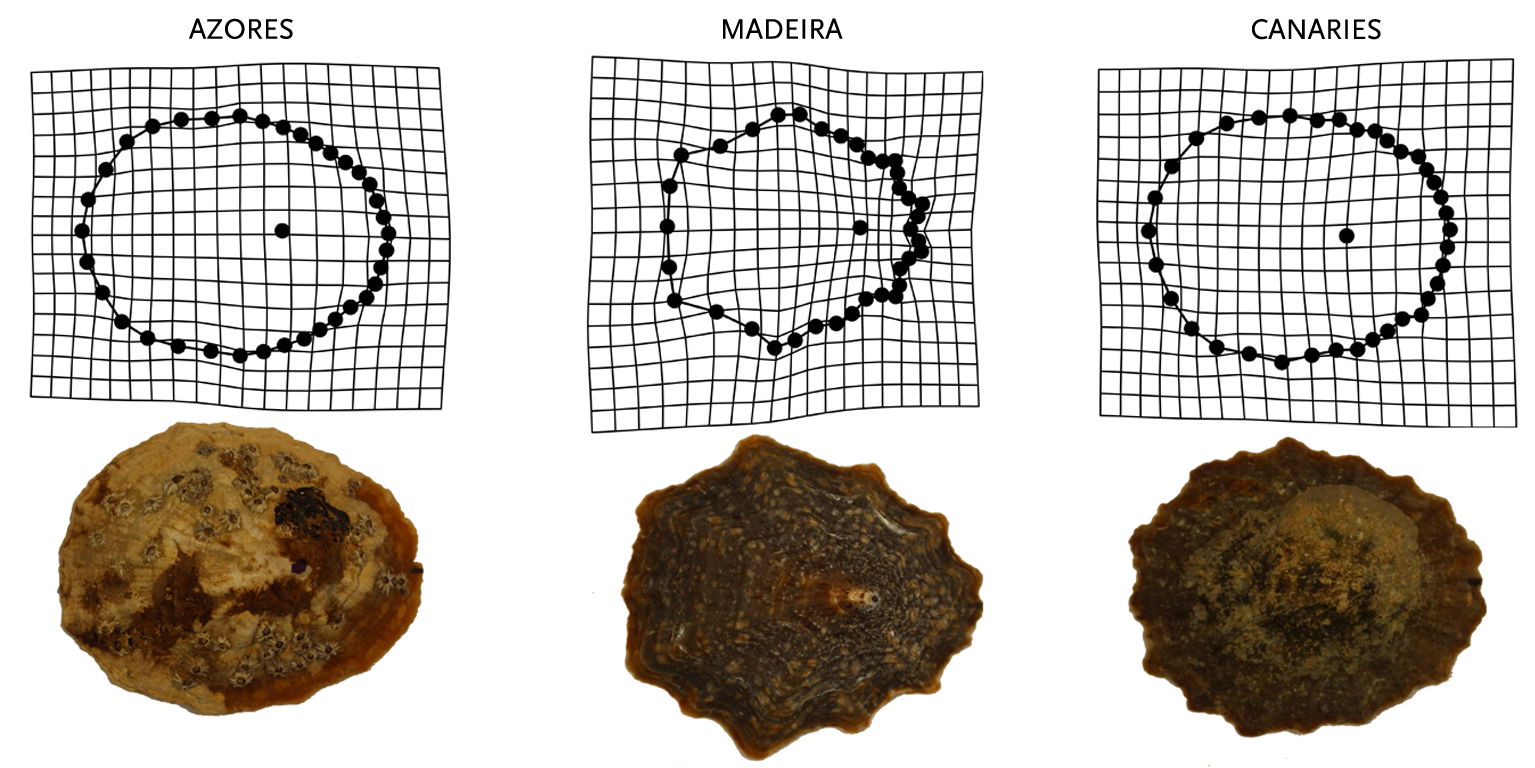
**

**Figure S5**. Individual shells of specimens identified as more similar/closest to each group mean shape. Thin-plate spline deformations grids are shown for each archipelago shape variation against overall mean shape.

**
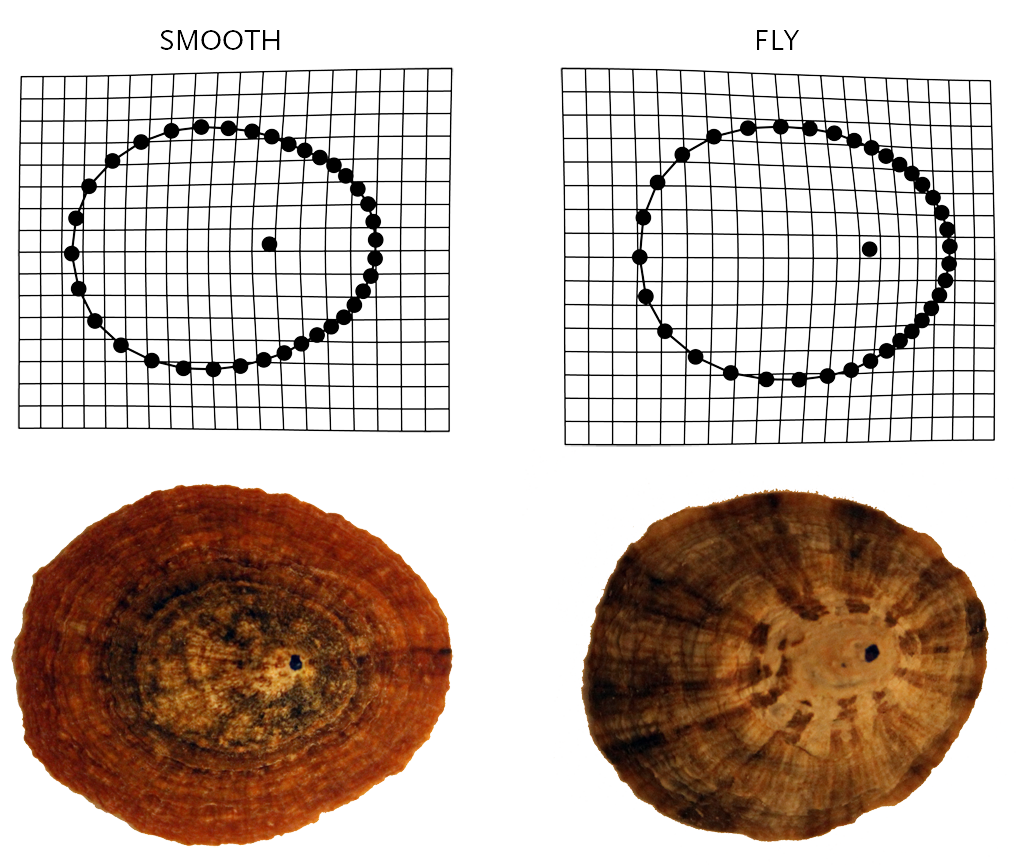
**

**Figure S6**. Individual shells of specimens identified as more similar/closest to each group mean shape. Thin-plate spline deformations grids are shown for each morphotypes (‘smooth’ and ‘fly’) shape variation against overall mean shape.

**Figure S7.** Correlation of pairwise estimates of F_ST_ and D_est_ between populations of *Patella candei* in NE Atlantic.

**Table S1.** A two-way PERMANOVA examining differences in shell length within archipelagos and islands.

|  | df | MS | *F* | |
| --- | --- | --- | --- | --- |
| ARCHIPELAGO | 2 | 40.259 | 1.2015 | |
| ISLAND (ARCHIPELAGO) | 9 | 43.914 | 127.09*** | |
| Residual | 905 | 0.34554 |  | |
| Total | 916 |  |  | |
| * P < 0.05. ** P < 0.01. *** P < 0.001 | | | |  |

**Table S2.** Correlation matrices between *P. candei* shell morphological variables.

|  | Distance measures | | | | |  | Morphometric descriptors | | | |
| --- | --- | --- | --- | --- | --- | --- | --- | --- | --- | --- |
|  | SL | SW | SWA | SAA | SH |  | BE | BEC | CO | CE |
| Shell length (SL) | 1.00 |  |  |  |  |  |  |  |  |  |
| Shell width (SW) | 0.99 | 1.00 |  |  |  |  |  |  |  |  |
| Shell width at apex (SWA) | 0.98 | 0.99 | 1.00 |  |  |  |  |  |  |  |
| Distance from apex to anterior tip (SAA) | 0.93 | 0.91 | 0.94 | 1.00 |  |  |  |  |  |  |
| Shell height (SH) | 0.77 | 0.77 | 0.82 | 0.91 | 1.00 |  |  |  |  |  |
|  |  |  |  |  |  |  |  |  |  |  |
| Base ellipticity (BE) | 0.39 | 0.53 | 0.49 | 0.36 | 0.36 |  | 1.00 |  |  |  |
| Base eccentricity (BEC) | 0.08 | 0.03 | 0.19 | 0.26 | 0.36 |  | -0.19 | 1.00 |  |  |
| Conicity (CO) | -0.08 | -0.06 | 0.02 | 0.22 | 0.55 |  | 0.12 | 0.46 | 1.00 |  |
| Cone eccentricity (CE) | 0.26 | 0.26 | 0.33 | 0.59 | 0.68 |  | 0.18 | 0.46 | 0.75 | 1.00 |

**Table S3**. A two-way PERMANOVA examining differences in shell conicity among archipelagos and islands.

|  | df | MS | *F* | *Components of variation (%)* |  |
| --- | --- | --- | --- | --- | --- |
| ARCHIPELAGO | 2 | 0.4294 | 7.3934* | 53 | |
| ISLAND (ARCHIPELAGO) | 9 | 7.5844E-2 | 51.737*** | 18 | |
| Residual | 905 | 1.4659E-3 |  | 29 | |
| Total | 916 |  |  |  | |
| *Pairwise comparison*s |  |  |  |  | |
| AZORES = CANARIES; AZORES ≠ MADEIRA; CANARIES ≠ MADEIRA | | | | | |
| * P < 0.05. ** P < 0.01. *** P < 0.001 | | | | | |

**Table S4**. A two-way PERMANOVA examining differences in shell base ellipticity among archipelagos and islands. Individual shell length was used as the covariate.

|  | df | MS | F | *Components of variation (%)* |
| --- | --- | --- | --- | --- |
| SL | 1 | 0.27965 | 25.097*** | 12 |
| ARCHIPELAGO | 2 | 7.2687E-2 | 5.0872* | 19 |
| ISLAND (ARCHIPELAGO) | 9 | 1.7081E-2 | 12.087*** | 9 |
| SL × ARCHIPELAGO | 2 | 2.9074E-3 | 2.0574 | 1 |
| SL × ISLAND (ARCHIPELAGO) | 9 | 2.2428E-3 | 1.5871 | 1 |
| Residual | 893 | 1.4131E-3 |  | 58 |
| Total | 916 |  |  |  |
| *Pairwise comparison*s |  |  |  |  |
| AZORES = CANARIES; AZORES ≠ MADEIRA; CANARIES ≠ MADEIRA | | | | |
| * P < 0.05. ** P < 0.01. *** P < 0.001 | | | | |

**Table S5**. Procrustes ANOVA examining differences in patterns of shell shape variation among archipelagos (10 000 random permutations) for SMALL and BIG datasets. Centroid size (CS) was used as a covariate.

| *Small limpets* | df | MS | F |
| --- | --- | --- | --- |
| SIZE *log(CS)* | 1 | 0.0044 | 1.630 |
| ARCHIPELAGO | 2 | 0.0083 | 3.055* |
| SIZE *log(CS)* × ARCHIPELAGO | 2 | 0.0029 | 1.064 |
| Total | 29 |  |  |
| *Pairwise comparisons* |  |  |  |
| AZORES = CANARIES; AZORES ≠ MADEIRA; CANARIES ≠ MADEIRA | | | |
| * P < 0.05. ** P < 0.01. *** P < 0.001 | | | |
|  | | | |
| *Big limpets* | df | MS | F |
| SIZE *log(CS)* | 1 | 0.0014 | 0.7670 |
| ARCHIPELAGO | 2 | 0.0093 | 5.0378** |
| SIZE *log(CS)* × ARCHIPELAGO | 2 | 0.0024 | 1.2970 |
| Total | 23 |  |  |
| *Pairwise comparisons* |  |  |  |
| AZORES = CANARIES; AZORES ≠ MADEIRA; CANARIES ≠ MADEIRA | | | |
| * P < 0.05. ** P < 0.01. *** P < 0.001 | | | |

**Table S6**. Procrustes ANOVA examining differences in patterns of shell shape variation among *P. candei* morphotypes in Azores (10 000 random permutations). Centroid size (CS) was used as a covariate. Slope pairwise comparisons among morphotypes are shown; contrasts in slope vector length and angles between slope vectors are shown in upper and lower diagonal, respectively.

|  | df | R^2^ | Z | F |
| --- | --- | --- | --- | --- |
| SIZE *log(CS)* | 1 | 0.281 | 11.185 | 26.605*** |
| MORPH | 1 | 0.081 | 4.309 | 7.686** |
| SIZE *log(CS)* × MORPH | 1 | 0.077 | 4.474 | 7.305** |
| Total | 56 |  |  |  |
|  |  |  |  |  |
| *Slope pairwise comparison* | FLY | SMOOTH |  |  |
| FLY | - | 4.334 ** |  |  |
| SMOOTH | 140.7 | - |  |  |
| * P < 0.05. ** P < 0.01. *** P < 0.001  Effect sizes (Z) are standard deviations of observed. | | | | |

**Table S7.** Genetic variation observed at twelve microsatellite loci within eleven populations sampled for *Patella candei* (see Fig. 1 for population codes).

|  | FLO | COR | FAI | PIC | SJO | GRA | TER | SMI | SMA | MAD | GCA | All populations |
| --- | --- | --- | --- | --- | --- | --- | --- | --- | --- | --- | --- | --- |
| N | 58 | 49 | 54 | 54 | 46 | 49 | 53 | 49 | 50 | 50 | 48 | 560 |
| CAN9 |  |  |  |  |  |  |  |  |  |  |  |  |
| Na | 9 | 10 | 11 | 10 | 10 | 9 | 10 | 9 | 10 | 0 | 0 | 12 |
| Ar(30) | 7.16 | 8.03 | 8.42 | 7.59 | 8.41 | 7.74 | 7.43 | 7.16 | 7.96 | - | - | - |
| Ap(30) | 0.17 | 0.22 | 0.32 | 0.19 | 0.17 | 0.00 | 0.08 | 0.00 | 0.15 | - | - | **-** |
| H_O_ | 0.672 | 0.755 | 0.704 | 0.704 | 0.739 | 0.830 | 0.774 | 0.694 | 0.755 | 0.000 | 0.000 | 0.602 |
| H_E_ | 0.808 | 0.832 | 0.856 | 0.816 | 0.843 | 0.834 | 0.783 | 0.817 | 0.792 | 0.000 | 0.000 | 0.671 |
| F_IS_ | **0.169** | 0.093 | **0.180** | **0.139** | **0.124** | 0.005 | 0.012 | 0.152 | 0.048 | NA | NA | **0.105** |
| Null | 0.07^a^ | 0.03 | 0.09^a^ | 0.06 | 0.06 | 0.01 | 0.02 | 0.06^a^ | 0.02 | - | - |  |
| CAN18 |  |  |  |  |  |  |  |  |  |  |  |  |
| Na | 18 | 14 | 18 | 13 | 12 | 14 | 14 | 16 | 16 | 5 | 5 | 22 |
| Ar(30) | 11.04 | 10.02 | 11.34 | 9.22 | 9.82 | 9.78 | 9.44 | 11.15 | 10.58 | 4.75 | 3.98 | - |
| Ap(30) | 0.59 | 0.12 | 0.59 | 0.35 | 0.01 | 0.44 | 0.02 | 0.12 | 0.41 | 0.48 | 0.00 | **-** |
| H_O_ | 0.537 | 0.510 | 0.520 | 0.469 | 0.543 | 0.511 | 0.462 | 0.500 | 0.600 | 0.242 | 0.577 | 0.497 |
| H_E_ | 0.841 | 0.871 | 0.888 | 0.837 | 0.882 | 0.860 | 0.849 | 0.872 | 0.866 | 0.666 | 0.546 | 0.816 |
| F_IS_ | **0.363** | **0.417** | **0.417** | **0.442** | **0.386** | **0.409** | **0.459** | **0.429** | **0.310** | **0.640** | **-0.058** | **0.401** |
| Null | 0.16^a^ | 0.19^a^ | 0.19^a^ | 0.19^a^ | 0.18^a^ | 0.19^a^ | 0.21^a^ | 0.19^a^ | 0.14^a^ | 0.26^a^ | 0.04^a^ |  |
| CAN23 |  |  |  |  |  |  |  |  |  |  |  |  |
| Na | 4 | 3 | 5 | 4 | 5 | 3 | 4 | 3 | 2 | 3 | 2 | 8 |
| Ar(30) | 2.52 | 2.52 | 3.18 | 2.76 | 3.35 | 2.31 | 2.78 | 2.67 | 2.00 | 2.20 | 1.78 | - |
| Ap(30) | 0.18 | 0.00 | 0.33 | 0.14 | 0.41 | 0.00 | 0.22 | 0.00 | 0.00 | 1.20 | 0.00 | - |
| H_O_ | 0.448 | 0.347 | 0.556 | 0.426 | 0.500 | 0.396 | 0.481 | 0.367 | 0.360 | 0.143 | 0.083 | 0.497 |
| H_E_ | 0.435 | 0.394 | 0.462 | 0.452 | 0.476 | 0.432 | 0.430 | 0.408 | 0.347 | 0.135 | 0.081 | 0.368 |
| F_IS_ | -0.031 | 0.120 | -0.206 | 0.058 | -0.051 | 0.085 | -0.119 | 0.101 | -0.039 | -0.057 | -0.033 | -0.016 |
| Null | 0.00 | 0.04 | 0.00 | 0.01 | 0.00 | 0.02 | 0.00 | 0.03 | 0.00 | 0.00 | 0.00 |  |
| CAN25 |  |  |  |  |  |  |  |  |  |  |  |  |
| Na | 6 | 6 | 9 | 8 | 7 | 7 | 7 | 7 | 8 | 5 | 5 | 14 |
| Ar(30) | 4.96 | 5.06 | 6.94 | 6.55 | 5.97 | 6.11 | 5.55 | 6.24 | 6.81 | 5.00 | 3.95 | - |
| Ap(30) | 0.00 | 0.00 | 0.73 | 0.00 | 0.00 | 0.00 | 0.10 | 0.00 | 0.12 | 1.00 | 1.32 | - |
| H_O_ | 0.483 | 0.563 | 0.327 | 0.404 | 0.333 | 0.356 | 0.481 | 0.487 | 0.489 | 0.133 | 0.000 | 0.369 |
| H_E_ | 0.630 | 0.720 | 0.757 | 0.715 | 0.747 | 0.736 | 0.686 | 0.761 | 0.799 | 0.453 | 0.389 | 0.672 |
| F_IS_ | **0.235** | **0.220** | **0.571** | **0.438** | **0.556** | **0.520** | **0.302** | **0.363** | **0.390** | **0.713** | **1.000** | **0.440** |
| Null | 0.11^a^ | 0.08^a^ | 0.24^a^ | 0.19^a^ | 0.23^a^ | 0.22^a^ | 0.13^a^ | 0.16^a^ | 0.17^a^ | 0.21^a^ | 0.31^a^ |  |
| CAN26 |  |  |  |  |  |  |  |  |  |  |  |  |
| Na | 5 | 5 | 5 | 4 | 5 | 4 | 3 | 4 | 4 | 0 | 0 | 6 |
| Ar(30) | 3.96 | 3.57 | 3.90 | 3.51 | 4.15 | 3.88 | 3.00 | 3.34 | 3.30 | - | - | - |
| Ap(30) | 0.15 | 0.33 | 0.03 | 0.00 | 0.09 | 0.02 | 0.00 | 0.00 | 0.00 | - | - | **-** |
| H_O_ | 0.621 | 0.510 | 0.365 | 0.388 | 0.419 | 0.426 | 0.510 | 0.341 | 0.375 | 0.000 | 0.000 | 0.360 |
| H_E_ | 0.616 | 0.583 | 0.609 | 0.545 | 0.633 | 0.605 | 0.639 | 0.574 | 0.534 | 0.000 | 0.000 | 0.485 |
| F_IS_ | -0.007 | 0.125 | **0.403** | **0.291** | **0.341** | **0.299** | 0.203 | **0.408** | **0.299** | NA | NA | **0.252** |
| Null | 0.02 | 0.02 | 0.14^a^ | 0.11^a^ | 0.14^a^ | 0.11^a^ | 0.08 | 0.16^a^ | 0.12^a^ | - | - |  |
| CAN27 |  |  |  |  |  |  |  |  |  |  |  |  |
| Na | 5 | 4 | 4 | 4 | 4 | 4 | 5 | 3 | 4 | 3 | 4 | 9 |
| Ar(30) | 3.22 | 3.08 | 3.21 | 3.11 | 3.25 | 3.34 | 3.31 | 2.67 | 2.61 | 2.31 | 3.06 | - |
| Ap(30) | 0.14 | 0.00 | 0.38 | 0.01 | 0.01 | 0.02 | 0.29 | 0.00 | 0.31 | 0.00 | 1.22 | - |
| H_O_ | 0.500 | 0.327 | 0.519 | 0.519 | 0.522 | 0.551 | 0.472 | 0.531 | 0.490 | 0.449 | 0.063 | 0.360 |
| H_E_ | 0.536 | 0.509 | 0.537 | 0.548 | 0.557 | 0.538 | 0.528 | 0.535 | 0.508 | 0.487 | 0.535 | 0.529 |
| F_IS_ | 0.067 | 0.361 | **0.034** | **0.055** | 0.065 | -0.025 | 0.108 | 0.008 | 0.037 | 0.080 | **0.884** | **0.148** |
| Null | 0.01 | 0.11^a^ | 0.04 | 0.05 | 0.00 | 0.00 | 0.06 | 0.00 | 0.00 | 0.02^a^ | 0.31 |  |
| CAN32 |  |  |  |  |  |  |  |  |  |  |  |  |
| Na | 5 | 4 | 4 | 4 | 4 | 5 | 4 | 5 | 5 | 12 | 10 | 18 |
| Ar(30) | 3.59 | 3.01 | 3.18 | 3.23 | 3.12 | 3.76 | 3.22 | 3.81 | 3.56 | 9.57 | 7.29 | - |
| Ap(30) | 0.28 | 0.07 | 0.06 | 0.00 | 0.00 | 0.12 | 0.00 | 0.21 | 0.13 | 4.09 | 1.24 | - |
| H_O_ | 0.333 | 0.163 | 0.259 | 0.352 | 0.239 | 0.383 | 0.327 | 0.400 | 0.347 | 0.723 | 0.638 | 0.379 |
| H_E_ | 0.336 | 0.225 | 0.310 | 0.363 | 0.305 | 0.337 | 0.347 | 0.349 | 0.367 | 0.849 | 0.700 | 0.408 |
| F_IS_ | 0.009 | 0.278 | 0.164 | 0.031 | 0.217 | -0.140 | 0.059 | -0.147 | 0.056 | 0.149 | 0.089 | **0.073** |
| Null | 0.00 | 0.08 | 0.05 | 0.02 | 0.06 | 0.00 | 0.03 | 0.00 | 0.03 | 0.04 | 0.00 |  |
| CAN33 |  |  |  |  |  |  |  |  |  |  |  |  |
| Na | 4 | 3 | 2 | 5 | 4 | 3 | 3 | 3 | 3 | 0 | 0 | 10 |
| Ar(30) | 2.63 | 2.26 | 1.73 | 2.59 | 2.60 | 2.06 | 1.78 | 1.83 | 1.96 | - | - | - |
| Ap(30) | 0.68 | 0.32 | 0.00 | 0.67 | 0.63 | 0.53 | 0.08 | 0.36 | 0.14 | - | - | **-** |
| H_O_ | 0.053 | 0.170 | 0.074 | 0.132 | 0.044 | 0.000 | 0.019 | 0.061 | 0.040 | 0.000 | 0.000 | 0.054 |
| H_E_ | 0.135 | 0.159 | 0.072 | 0.127 | 0.129 | 0.082 | 0.057 | 0.060 | 0.078 | 0.000 | 0.000 | 0.082 |
| F_IS_ | **0.612** | -0.071 | -0.029 | -0.039 | **0.657** | **1.000** | **0.665** | -0.014 | 0.491 | NA | NA | **0.340** |
| Null | 0.12^a^ | 0.00 | 0.00 | 0.00 | 0.13^a^ | 0.14^a^ | 0.09^a^ | 0.00 | 0.08 | - | - |  |
| CAN40 |  |  |  |  |  |  |  |  |  |  |  |  |
| Na | 12 | 8 | 11 | 9 | 10 | 9 | 9 | 10 | 8 | 7 | 6 | 17 |
| Ar(30) | 6.59 | 6.10 | 7.48 | 6.61 | 5.90 | 5.81 | 4.86 | 6.86 | 5.20 | 5.98 | 5.59 | - |
| Ap(30) | 0.16 | 0.12 | 0.05 | 0.03 | 0.47 | 0.05 | 0.01 | 0.02 | 0.31 | 1.32 | 1.13 | - |
| H_O_ | 0.466 | 0.449 | 0.463 | 0.385 | 0.261 | 0.457 | 0.327 | 0.447 | 0.367 | 0.148 | 0.194 | 0.360 |
| H_E_ | 0.612 | 0.542 | 0.654 | 0.616 | 0.521 | 0.605 | 0.424 | 0.646 | 0.554 | 0.663 | 0.748 | 0.599 |
| F_IS_ | **0.241** | 0.174 | **0.294** | **0.378** | **0.502** | **0.248** | **0.230** | **0.311** | **0.339** | **0.780** | **0.743** | **0.368** |
| Null | 0.08^a^ | 0.02 | 0.08^a^ | 0.13^a^ | 0.16^a^ | 0.09^a^ | 0.08^a^ | 0.09^a^ | 0.13^a^ | 0.31 | 0.32^a^ |  |
| CAN53 |  |  |  |  |  |  |  |  |  |  |  |  |
| Na | 4 | 5 | 5 | 4 | 7 | 5 | 5 | 5 | 4 | 6 | 4 | 9 |
| Ar(30) | 2.46 | 3.63 | 3.40 | 2.52 | 4.60 | 3.77 | 3.40 | 3.88 | 3.40 | 4.55 | 3.44 | - |
| Ap(30) | 0.00 | 0.00 | 0.19 | 0.00 | 0.46 | 0.21 | 0.28 | 0.00 | 0.00 | 0.35 | 0.00 | - |
| H_O_ | 0.155 | 0.306 | 0.222 | 0.185 | 0.370 | 0.245 | 0.302 | 0.306 | 0.245 | 0.633 | 0.292 | 0.296 |
| H_E_ | 0.176 | 0.308 | 0.224 | 0.203 | 0.409 | 0.279 | 0.313 | 0.326 | 0.308 | 0.563 | 0.266 | 0.599 |
| F_IS_ | 0.119 | 0.005 | 0.006 | 0.089 | 0.097 | 0.122 | 0.036 | 0.061 | 0.206 | -0.124 | -0.099 | 0.035 |
| Null | 0.03 | 0.00 | 0.00 | 0.03 | 0.04 | 0.04 | 0.00 | 0.03 | 0.06 | 0.00 | 0.00 |  |
| CAN56 |  |  |  |  |  |  |  |  |  |  |  |  |
| Na | 2 | 3 | 2 | 4 | 2 | 2 | 3 | 2 | 3 | 2 | 1 | 6 |
| Ar(30) | 1.26 | 2.17 | 1.63 | 2.29 | 1.33 | 1.68 | 1.58 | 1.31 | 1.83 | 1.37 | 1.00 | - |
| Ap(30) | 0.26 | 0.21 | 0.02 | 0.18 | 0.01 | 0.02 | 0.16 | 0.16 | 0.04 | 0.37 | 0.00 | - |
| H_O_ | 0.018 | 0.083 | 0.019 | 0.074 | 0.022 | 0.063 | 0.038 | 0.020 | 0.061 | 0.024 | 0.000 | 0.038 |
| H_E_ | 0.018 | 0.120 | 0.055 | 0.107 | 0.022 | 0.061 | 0.038 | 0.020 | 0.060 | 0.024 | 0.000 | 0.048 |
| F_IS_ | NA | 0.305 | 0.662 | 0.313 | NA | -0.022 | -0.005 | NA | -0.014 | NA | NA | **0.205** |
| Null | 0.00 | 0.07 | 0.09^a^ | 0.07 | 0.00 | 0.00 | 0.00 | 0.00 | 0.00 | 0.00 | 0.00 |  |
| CAN60 |  |  |  |  |  |  |  |  |  |  |  |  |
| Na | 3 | 3 | 2 | 3 | 3 | 3 | 2 | 2 | 2 | 5 | 3 | 7 |
| Ar(30) | 2.25 | 2.52 | 2.00 | 2.46 | 2.54 | 2.50 | 1.94 | 1.97 | 1.95 | 3.80 | 2.32 | - |
| Ap(30) | 0.13 | 0.11 | 0.00 | 0.36 | 0.12 | 0.12 | 0.00 | 0.00 | 0.00 | 1.49 | 0.00 | - |
| H_O_ | 0.086 | 0.000 | 0.056 | 0.093 | 0.022 | 0.085 | 0.113 | 0.063 | 0.041 | 0.104 | 0.043 | 0.064 |
| H_E_ | 0.242 | 0.282 | 0.268 | 0.218 | 0.282 | 0.196 | 0.141 | 0.172 | 0.151 | 0.475 | 0.390 | 0.256 |
| F_IS_ | **0.646** | **1.000** | **0.794** | **0.577** | **0.924** | **0.568** | 0.198 | **0.639** | **0.733** | **0.783** | **0.892** | **0.746** |
| Null | 0.16^a^ | 0.26^a^ | 0.20^a^ | 0.12^a^ | 0.24^a^ | 0.14^a^ | 0.05 | 0.14^a^ | 0.15^a^ | 0.27^a^ | 0.26^a^ |  |
| Multilocus |  |  |  |  |  |  |  |  |  |  |  |  |
| Mean Ar(30) | 4.30 | 4.33 | 4.70 | 4.37 | 4.59 | 4.40 | 4.02 | 4.41 | 4.26 | 4.39 | 3.60 | - |
| Mean Ap(30) | 0.26 | 0.16 | 0.30 | 0.18 | 0.22 | 0.16 | 0.11 | 0.14 | 0.16 | 1.14 | 0.55 | - |
| Mean H_O_ | 0.364 | 0.349 | 0.340 | 0.344 | 0.335 | 0.358 | 0.359 | 0.351 | 0.348 | 0.217 | 0.157 | 0.320 |
| Mean H_E_ | 0.449 | 0.462 | 0.474 | 0.462 | 0.484 | 0.464 | 0.436 | 0.462 | 0.447 | 0.360 | 0.304 | 0.437 |
| F_IS_ | **0.194** | **0.243** | **0.266** | **0.222** | **0.309** | **0.247** | **0.170** | **0.183** | **0.229** | **0.319** | **0.419** | **0.253** |
| *N* = number of samples; *Na* = number of alleles; *Ar(g)* = allelic richness (*g* accounts for the maximum standardized sample size i.e. twice the number of genotypes); *Ap(g)* = private allelic richness; *H*_O_ = observed heterozygosity; *H_E_* = unbiased expected heterozygosity; *F_IS_* = inbreeding coefficient. ^a^Microchecker detection for null alleles. Significant departure from Hardy–Weinberg equilibrium after False Discovery Rate correction is shown in bold. | | | | | | | | | | | | |

**Table S8**. Percentage of missing data for each locus across populations (see Fig.1 for population codes).

|  | FLO | COR | FAI | PIC | SJO | GRA | TER | SMI | SMA | MAD | GCA |
| --- | --- | --- | --- | --- | --- | --- | --- | --- | --- | --- | --- |
| CAN9 | 0 | 0 | 0 | 0 | 0 | 0 | 0 | 0 | 0 | 100 | 100 |
| CAN18 | 6 | 0 | 7 | 9 | 0 | 0 | 0 | 10 | 10 | 28 | 45 |
| CAN23 | 0 | 0 | 0 | 0 | 0 | 0 | 0 | 0 | 0 | 0 | 0 |
| CAN25 | 0 | 0 | 0 | 0 | 0 | 8 | 0 | 10 | 20 | 70 | 0 |
| CAN26 | 0 | 0 | 0 | 9 | 6 | 0 | 7 | 16 | 0 | 100 | 100 |
| CAN27 | 0 | 0 | 0 | 0 | 0 | 0 | 0 | 0 | 0 | 0 | 0 |
| CAN32 | 0 | 0 | 0 | 0 | 0 | 0 | 0 | 8 | 0 | 6 | 0 |
| CAN33 | 0 | 0 | 0 | 0 | 0 | 0 | 0 | 0 | 0 | 100 | 100 |
| CAN40 | 0 | 0 | 0 | 0 | 0 | 6 | 0 | 0 | 0 | 46 | 25 |
| CAN53 | 0 | 0 | 0 | 0 | 0 | 0 | 0 | 0 | 0 | 0 | 0 |
| CAN56 | 0 | 0 | 0 | 0 | 0 | 0 | 0 | 0 | 0 | 18 | 35 |
| CAN60 | 0 | 0 | 0 | 0 | 0 | 0 | 0 | 0 | 0 | 0 | 0 |

**Table S9**. Single-locus and multilocus F_ST_ (before and after the ENA correction method). and Jost’s D_est_ estimates for *Patella candei.*

| locus | F_ST_ | ^1^F_ST_ | D_est_ |
| --- | --- | --- | --- |
| CAN9 | 0.001 | 0.002 | **-** |
| CAN18 | 0.061 | 0.054 | 0.436 |
| CAN23 | 0.285 | 0.283 | 0.242 |
| CAN25 | 0.130 | 0.097 | 0.401 |
| CAN26 | 0.006 | 0.007 | - |
| CAN27 | 0.070 | 0.058 | 0.092 |
| CAN32 | 0.259 | 0.245 | 0.250 |
| CAN33 | 0.000 | 0.018 | - |
| CAN40 | 0.036 | 0.032 | 0.080 |
| CAN53 | 0.019 | 0.017 | 0.009 |
| CAN56 | 0.005 | 0.030 | 0.001 |
| CAN60 | 0.438 | 0.321 | 0.283 |
| All loci | 0.116 | 0.101 | 0.199 |
| 95% CI | (0.047-0.192) | (0.039-0.167) | (0.194-0.204) |

**Table S10**. Posterior probabilities of migration for individuals identified with mixed migrant ancestry. The notation [*i*. *j*] indexes the population source *i* and generation *j* (0=non-migrant. 1=1^ST^ generation migrant. 2=2^ND^ generation migrant) of migrant ancestry.

| Source population  *Individual* | CANARIES  *CE331* | | |  | MADEIRA  *CD318* | | |  | MADEIRA  *CD335* | | |
| --- | --- | --- | --- | --- | --- | --- | --- | --- | --- | --- | --- |
| Migrant ancestry | [0.0] | [1.0] | [2.0] |  | [0.0] | [1.0] | [2.0] |  | [0.0] | [1.0] | [2.0] |
|  | 0.000 | 0.000 | 0.747 |  | 0.000 | 0.871 | 0.000 |  | 0.000 | 0.352 | 0.000 |
|  | [0.1] | [1.1] | [2.0] |  | [0.1] | [1.1] | [2.0] |  | [0.1] | [1.1] | [2.0] |
|  | 0.000 | 0.239 | 0.000 |  | 0.000 | 0.000 | 0.001 |  | 0.000 | 0.000 | 0.212 |
|  | [0.2] | [1.2] | [2.2] |  | [0.2] | [1.2] | [2.2] |  | [0.2] | [1.2] | [2.2] |
|  | 0.000 | 0.014 | 0.000 |  | 0.000 | 0.000 | 0.128 |  | 0.000 | 0.000 | 0.435 |
